# Topography of Thalamic Projections Requires Attractive and Repulsive Functions of Netrin-1 in the Ventral Telencephalon

**DOI:** 10.1371/journal.pbio.0060116

**Published:** 2008-05-13

**Authors:** Ashton W Powell, Takayuki Sassa, Yongqin Wu, Marc Tessier-Lavigne, Franck Polleux

**Affiliations:** 1 Neuroscience Center, Department of Pharmacology, University of North Carolina Chapel Hill, Chapel Hill, North Carolina, United States of America; 2 Curriculum in Neuroscience, University of North Carolina Chapel Hill, Chapel Hill, North Carolina, United States of America; 3 Genentech, South San Francisco, California, United States of America; University of California, San Diego, United States of America

## Abstract

Recent studies have demonstrated that the topography of thalamocortical (TC) axon projections is initiated before they reach the cortex, in the ventral telencephalon (VTel). However, at this point, the molecular mechanisms patterning the topography of TC projections in the VTel remains poorly understood. Here, we show that a long-range, high-rostral to low-caudal gradient of Netrin-1 in the VTel is required in vivo for the topographic sorting of TC axons to distinct cortical domains. We demonstrate that Netrin-1 is a chemoattractant for rostral thalamic axons but functions as a chemorepulsive cue for caudal thalamic axons. In accordance with this model, *DCC* is expressed in a high-rostromedial to low-caudolateral gradient in the dorsal thalamus (DTh), whereas three *Unc5* receptors (*Unc5A–C*) show graded expression in the reverse orientation. Finally, we show that DCC is required for the attraction of rostromedial thalamic axons to the Netrin-1–rich, anterior part of the VTel, whereas DCC and Unc5A/C receptors are required for the repulsion of caudolateral TC axons from the same Netrin-1–rich region of the VTel. Our results demonstrate that a long-range gradient of Netrin-1 acts as a counteracting force from ephrin-A5 to control the topography of TC projections before they enter the cortex.

## Author Summary

The functional properties of each structure in the central nervous system are critically dependent on the precision of neuronal connectivity. The cerebral cortex in particular is a highly organized structure divided into many distinct cortical areas underlying important sensory, motor, and cognitive functions in the brain. Each primary cortical area receives its synaptic inputs from the periphery via the dorsal thalamus. The main relay station for sensory information to the cortex, the thalamus, can be divided into specific nuclei projecting topographically to individual cortical areas. How is the complex topography of thalamic axon projection to individual cortical areas specified during development? Recent evidence demonstrated that thalamic axons are routed to different cortical domains before they enter the cortex, by putative axon guidance cues present in the ventral forebrain. In the present study, we provide evidence that a secreted axon guidance cue, Netrin-1, expressed in a long-range gradient in the ventral forebrain, plays a critical role in the establishment of the topography of thalamic projections by directing different subsets of axons to specific cortical domains. These results provide important insights into the molecular mechanisms responsible for shaping the topographical patterns of thalamocortical axon projections in mammals.

## Abstract 3

A long-range gradient of Netrin-1 plays a critical role in the specification of the topography of thalamocortical projections in the ventral telencephalon. The function of Netrin-1 requires both its attractive and repulsive functions to guide different subsets of thalamic axons to specific cortical domains.

## Introduction

In the central nervous system, the vast majority of axonal projections are organized topographically. The dorsal thalamus (DTh) is a pivotal forebrain structure, receiving sensory inputs from the periphery and communicating with the cerebral cortex via thalamocortical (TC) axons. Each thalamic nucleus projects topographically to a unique set of cortical areas (interareal, first-order level of topography), and subsequently, axons emerging within a given thalamic nucleus establish a topographic map of a given sensory modality within each cortical area (intra-areal, second-order level of topography). Numerous anatomical studies have demonstrated that the *interareal* topography of TC projections is organized so that rostromedial thalamic neurons project to more-rostral cortical areas than caudolateral nuclei, which tend to project to more-caudal cortical areas [1–3]. The developmental mechanisms leading to the initial guidance and topographic sorting of TC axons, first in the ventral telencephalon (VTel) and, ultimately, in the dorsal telencephalon (or cortex), are still poorly understood at the molecular level (reviewed in [4,5]).

Previous results suggested that the precise topography characterizing TC projections arises from the progressive sorting of axons by a series of cues present along their pathway rather than by those exclusively present in their final target, the cortex [4,5]. First, analysis of mouse knockouts for genes patterning the ventral telencephalon, including *Ebf1* and *Dlx1/2*, revealed a severe disruption of the topography of TC axon projections [6]. Second, genetic manipulation of rostral patterning molecules such as FGF8 affects the relative positioning of cortical areas without initially changing the topography of TC projections to the appropriate “cortical domain” [7,8]. These findings suggested a model in which TC axons are guided to their appropriate cortical domain by extracortical cues, i.e., *before* reaching the cortex. Interestingly, at later stages, unidentified cortical cues are able to redirect thalamic axon outgrowth to the appropriate cortical area inside the cortex proper [8], a result also found in heterotopic cortical grafting experiments [9].

Using a novel in vitro assay (the “whole-mount telencephalic” assay), we have demonstrated that axons originating from different rostrocaudal domains of the DTh respond differentially to topographic cues present in the VTel that guide these axons to specific cortical domains [10]. Currently, the only axon guidance cue identified to play a role in the topographic sorting of TC axons is *ephrin-A5*, which is expressed in a high-caudal to low-rostral gradient in the VTel [11]. In a complementary fashion, several EphA receptors, including *EphA4*, *EphA3*, and *EphA7*, are expressed in high-rostromedial to low-caudolateral gradients in the DTh [11]. Using the whole-mount telencephalic assay developed by Seibt et al. [10], a parallel study demonstrated that the graded expression of *ephrin-A5* in the VTel and some of its receptors such as *EphA7* and *EphA4* in the DTh play a role in the topographic sorting of TC axons in the VTel. Interestingly, ephrin-A5–EphA4 double-knockout (dKO) mice show a significant and fully penetrant topographic shift of TC projections at the level of the VTel, leading to the misprojection of some thalamic motor axons to aberrantly more-caudal areas such as the primary somatosensory cortex. However, TC axon projections still display a significant level of topography in the ephrin-A5–EphA4 dKO mouse [11], suggesting the existence of other axon guidance cues involved in the topographic sorting of TC axons in the VTel [5]. This idea is reinforced by the fact that the caudal outgrowth of TC axons in the VTel is not easily explained simply by the lack of responsiveness of EphA-deficient caudal thalamic axons to caudally enriched ephrin-A5 [11].

In the present study, we demonstrate that *Netrin-1* is expressed in a high-rostral to low-caudal long-range gradient within the VTel. Using a novel quantitative axon tracing technique with high spatial resolution, we show that *Netrin-1–*deficient embryos show a severe disruption of the topography of TC projections at the level of VTel *before* they enter the cortex. Interestingly, both rostral and caudal thalamic axons are affected in the *Netrin-1* knockout mouse, and we further demonstrate that both the attractive and repulsive functions of Netrin-1 are required for proper topographic projections of TC axons along the anteroposterior axis of the VTel. These results (1) provide new insights into the molecular and cellular mechanisms specifying the topography of TC axons and (2) demonstrate that the secreted ligand Netrin-1 can specify the topography of projection of large ensembles of axons, a function almost exclusively attributed to the membrane-bound ephrin/Eph signaling system [12] and more recently to another set of secreted cues (Wnt) and their receptor Ryk [13].

## Results

### A Novel Quantitative Method to Trace and Reconstruct the Topography of Thalamocortical Projections In Vivo

**Figure 1:**
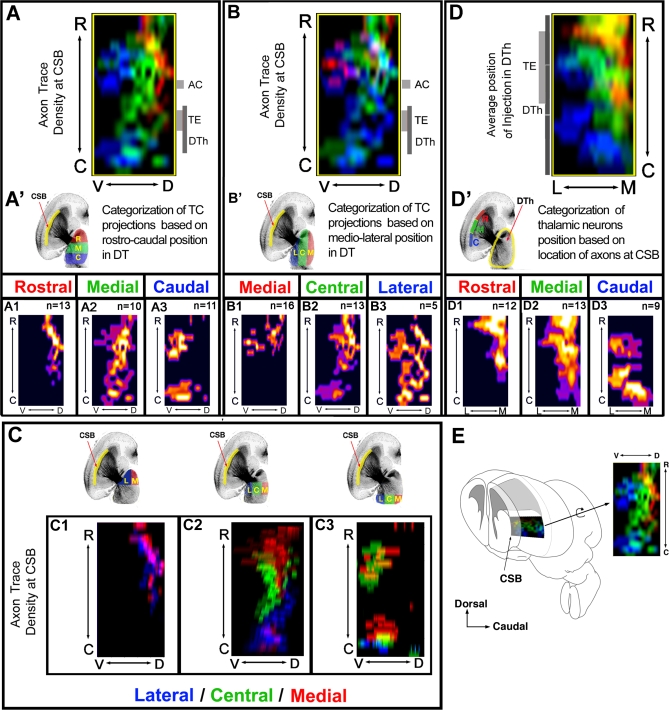
Precise Topography of Thalamocortical Projections Achieved at the Level of the Ventral Telencephalon Before Entering the Cortex (A) Averaged axon density maps quantified from multiple BDA injections (*n* numbers in [A1–A3]) clustered in three, arbitrarily defined thirds along the rostrocaudal axis of the E18.5 mouse DTh (red indicates rostral; green, medial; and blue, caudal; as shown in [A′]). (A1–A3) Individual average axon density maps for thalamic injections clustered in the rostral- (A1), medial- (A2), or caudal-most (A3) third of the DTh. (B) Averaged axon density maps quantified from multiple BDA injections clustered along the mediolateral axis of the DTh (red indicates medial; green, central; and blue, lateral; as shown in [B′]). (C) Averaged axon density maps shown in (A1) (rostral-most third of DT split in lateral and medial halves), (A2) (medial third along rostrocaudal extent), and (A3) (caudal third along rostrocaudal extent) were further subdivided into halves (C1) or thirds (C2 and C3) along the mediolateral axis. This analysis demonstrates the topographic segregation of thalamic axon projections before they enter the cortex at E18.5. (D and D′) Averaged position of BDA injection sites in the DTh leading to axons crossing CSB at its rostral- (red), medial- (green), or caudal-most (blue) third. This 2-D map represents a dorsal view of the DTh, compressed along its dorsoventral axis. (D1–D3) Individual averaged density maps of thalamic injection sites leading to axons crossing the CSB at its rostral- (D1), medial- (D2), or caudal-most (D3) third. (E) Schematic representation of the anatomical location of our 2-D, averaged axon density maps shown in this figure as well as Figures 4, S3, and S5. C, caudal; D, dorsal; R, rostral; V, ventral.

The introduction of fluorescent carbocyanine dyes as axonal tracers represented a technical breakthrough in our ability to map the development of neuronal connectivity, especially at embryonic stages in rodents [14]. However, fluorescent carbocyanine dyes such as DiI present several important limitations, including long diffusion time, imcompatibility with immunofluorescent techniques, and most importantly, significant diffusion at the site of injection. In order to circumvent most of these problems, we adapted a well-established anterograde axon tracing technique using lysine-fixable, low molecular weight biotinylated dextran amine (BDA, or biocytin) microinjection in the DTh of embryonic mouse [15,16]. Using microinjections of BDA in the DTh of mouse embryos (Figure S1A) enables high spatial resolution (few hundreds neurons labeled; Figure 1B′–1D) and is fully compatible with immunofluorescence (Figure S1B–1D). Complete anterograde filling of the axon is achieved over long distances (1–2 mm) within only 4–6 h following BDA injection, as shown by the presence of large growth cones at the tip of the majority of axons (Figure S1E).

**Figure 4:**
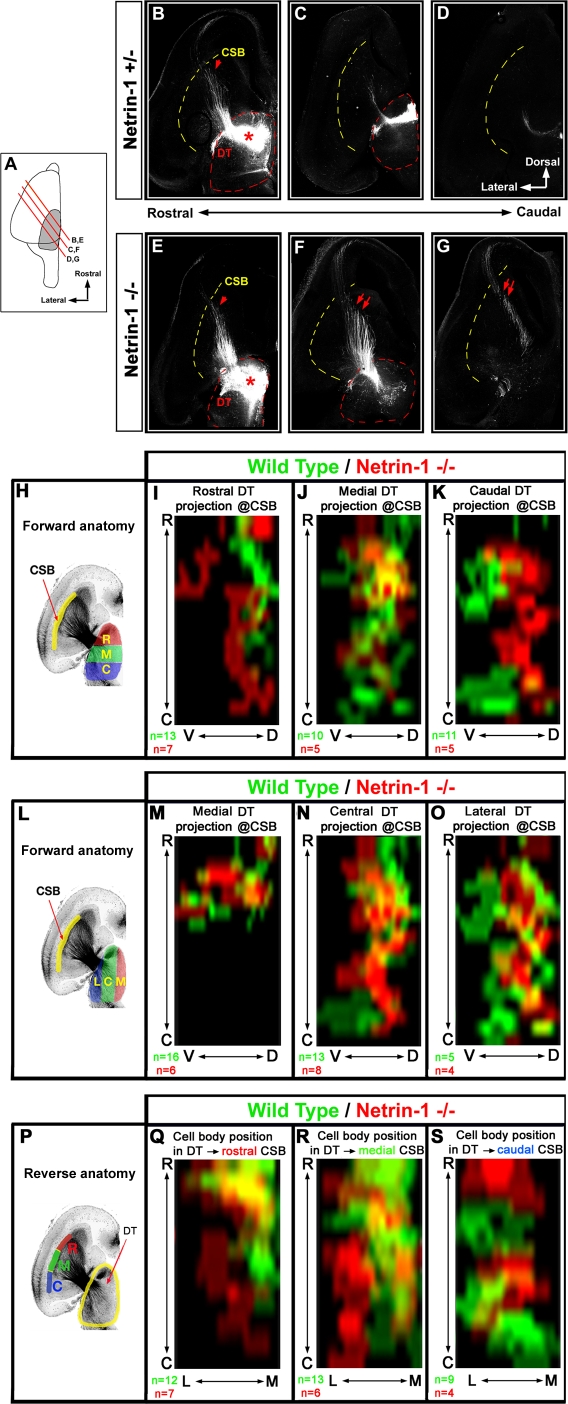
Netrin-1 Is Necessary for the Establishment of the Topography of Thalamocortical Projections in the Ganglionic Eminence In Vivo (A) Diagram of the level of the oblique sections used to visualize TC projections in (B–G). (B–G) Injections of BDA in the rostral third of the DTh (DT) of control (wild-type *Netrin-1^+/+^*; [B–D]) or *Netrin-1* knockout (*Netrin-1* ^−/−^) E18.5 embryos (E–G) reveal that thalamic axons originating from the rostral-most part of the DTh project more caudally (arrows in [F and G]) in the VTel of *Netrin-1* knockout than in control embryos. (H–O) Averaged axon density maps showing the distribution of thalamic axons at the CSB of E18.5 wild-type (green) or *Netrin-1* ^−/−^ embryos (red) for thalamic injections clustered along the rostromedial axis ([I–K]; as depicted in [H]) or the mediolateral axis ([M–O]; as depicted in [L]). (P–S) Averaged density maps of injection sites in the DTh leading to thalamic axons crossing the CSB at the rostral- (Q), medial- (R), or caudal-most (S) third of the CSB in the wild-type control mice (green) or *Netrin-1* ^−/−^ at E18.5. For statistical analysis of these density maps, see Figure S6. C, caudal; D, dorsal. R, rostral; V, ventral.

In order to normalize, register, and quantify the topography of TC axon projections in the VTel from microinjections performed in multiple mouse embryos, we developed a series of quantitative tools using axon tracing and image analysis allowing (1) reconstruction of the size and position of the BDA injection site in the DTh (Figure S1G, S1I, S1K, and S1M), as well as (2) tracing of thalamic axon projections throughout the telencephalon (Figure S1F, S1H, S1J, and S1L), and (3) quantitative analysis of the mapping of TC projections resulting from multiple injections in a large number of individuals. In order to best represent the degree of topographic sorting achieved by thalamic axons in the VTel (i.e., just before entering the cortex), we chose an anatomical landmark lying at the interface between the ventral and the dorsal telencephalon: the corticostriatal boundary (CSB; also known as the pallial–subpallial boundary; Figures 1E, S1P, S1R, and S2). The axon density maps used throughout this study (as shown in Figure S1S) is a flat, 2-D representation of the CSB as viewed by a virtual observer looking at the telencephalon from a lateral perspective (see Figure 1E).

### Topography of Thalamocortical Projections Established in the Ventral Telencephalon

Using this approach, we precisely and quantitatively mapped the organization of the axonal projections originating from different regions of the DTh at the level of the VTel. To do this, we performed a series of random microinjections of BDA in the DTh of E15.5 (Figure S3), when TC axons are still pioneering the VTel en route to the cortex, and E18.5 mouse embryos, a stage when all thalamic axons have reached the cortex [17]. Only injections representing less than 5% of the total volume of the DTh were analyzed in order to ensure that small groups of thalamic neurons are labeled, thus maintaining high spatial resolution.

Our results show that at E18.5, thalamic axons are highly segregated at the CSB according to their origin along two main axes of the DTh: the rostrocaudal axis (Figure 1A–1A3) and the mediolateral axis (Figure 1B–1B3 and 1C–1C3). Axons originating from the rostral third of the DTh cross the CSB (and therefore enter the cortex) at a more-rostral level (Figure 1A1) than axons originating from progressively more-caudolateral levels of the DTh (Figure 1A2 and 1A3). The same segregation is found for axons originating at different levels of the mediolateral axis of the DTh: axons originating from the medial third of the DTh reach the CSB at more-rostrodorsal levels (Figure 1C, 1C1, and 1B–1B3) than axons originating from progressively more-lateral thalamic domains (Figure 1C2, 1C3, and 1B–1B3), which cross the CSB at progressively more-caudal levels.

A converse way to represent the topography of thalamic axon projections in the VTel is to categorize thalamic axon populations based on where they cross the CSB and ask where they originate within the DTh (Figure 1D–1D3). This “reverse anatomy” approach reveals that axons crossing the CSB at rostral levels originate from more-rostromedial levels of the DTh (Figure 1D1) than axons crossing the caudal CSB, which originate from progressively more-caudolateral levels of the DTh (Figure 1D2 and 1D3).

Interestingly, the general topography of TC projections is already present at E15.5, as demonstrated using the same analysis (see Figure S3), confirming that the topographic sorting of TC axons is controlled by axon guidance cues present in the VTel when TC axons pioneer this intermediate target while forming the internal capsule (E14–E15; [10]).

These results (1) confirm previous studies that have primarily explored the organization of thalamic projections along the mediolateral axis of the DTh [6,7,10,18,19], (2) reveal that TC projections are also organized along the rostrocaudal axis as proposed previously [3,10,11,20], and therefore (3) that, most importantly, the overall axis of topography of TC projections is rostromedial to caudolateral [5]; (4) this new quantitative tool provides for the first time, to the best of our knowledge, a framework for the *quantitative* analysis of the function of axon guidance cues in the specification of the topography of TC projections in vivo.

### The Rostral Part of the Ganglionic Eminence Contains a Chemoattractive Cue for Rostral Thalamic Axons

**Figure 2:**
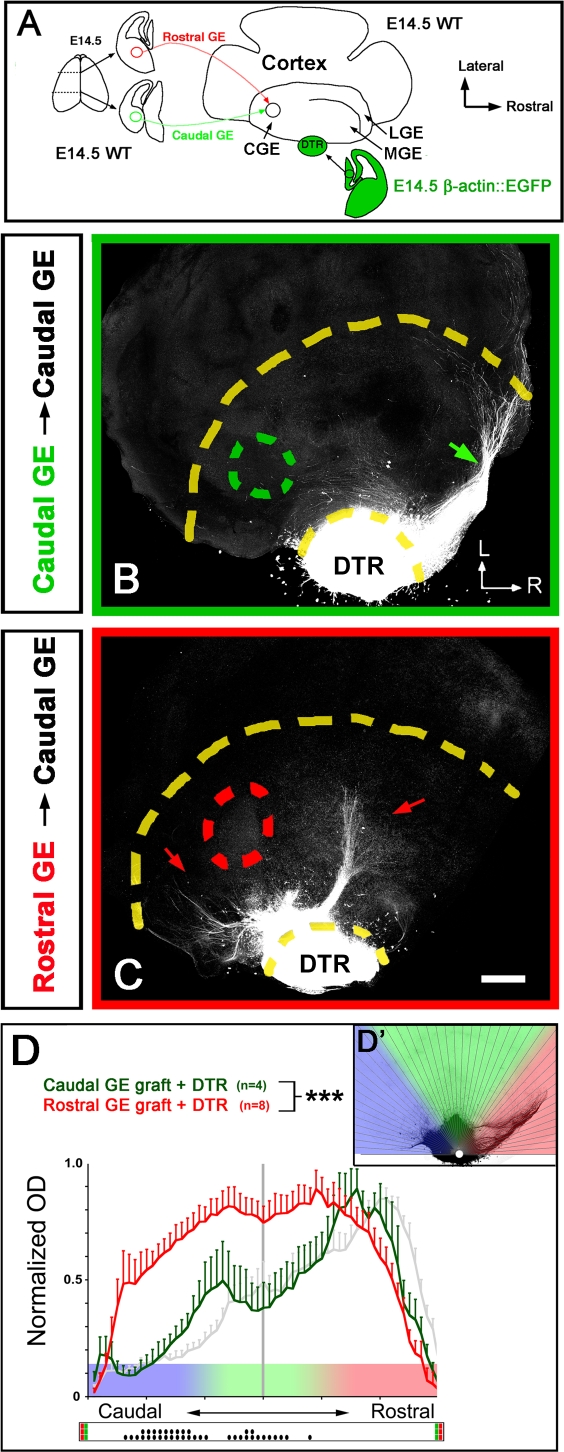
The Rostral Part of the Ganglionic Eminence Contains a Chemoattractant for Rostral Thalamic Axons (A) Rostral (red) or caudal (green) explants isolated from the mantle region of the ganglionic eminence (VTel) were isolated from 250-μm–thick vibratome sections and grafted into the caudal part of the VTel of a recipient E14.5 whole-mount telencephalic vesicle as described previously (Seibt et al., 2003 [10]). The rostral part of the DTh (DTR) isolated from coronal slices of an isochronic β-actin::EGFP-expressing mouse embryo (see Figure S11 for details on explant isolation) is cocultured with the whole-mount telencephalon for 4 d in vitro (div; see Materials and Methods for details). CGE, caudal ganglionic eminence; GE, ganglionic eminence; LGE, lateral ganglionic eminence; MGE, medial ganglionic eminence; WT, wild type. (B) Homotopic grafting (caudal VTel into caudal VTel) results in a normal outgrowth of rostral thalamic axons into the rostral domain of the VTel (arrow). L, lateral; R, rostral. (C) In contrast, heterotopic grafting (rostral VTel into the caudal VTel) results in a pronounced change in the topography of DTR axon projections, which invade more-caudal territories (red arrows) than in control grafts (see [B]). (D) Quantification of the topography of rostral thalamic axon outgrowth in the VTel presenting homotopic (green) or heterotopic (red) VTel graft into the caudal VTel. The gray curve illustrates the topography of DTR axons projection in control, nongrafted, experiments shown in Figure 5D. Each histogram represents the average normalized optical density (OD) from the EGFP signal measured in 60 radial bins centered on the thalamic explant as shown in (D′). Triple asterisks (***) indicate *p* < 0.001, ANOVA one-way test (overall effect: bins versus experimental conditions). The raster-like dot plot presented under the histograms represents the significance of individual bin comparisons between the two experimental conditions according to a Fisher Protected Least Significant Difference (PLSD) post hoc test (a single dot [•] indicates *p* < 0.05; and double dots [••] indicate *p* < 0.01). Scale bars in (B and C) represent 250 μm.

These results reveal that TC axons are organized in a precise “canvas” at the CSB as a consequence of axon guidance mechanisms specifying the topography of TC projections in the VTel, i.e., before they enter the dorsal telencephalon [4,5]. In order to identify some of the axon guidance cues patterning the topography of TC axon projections in the VTel, we used a whole-mount telencephalic assay recapitulating in vitro some of the key aspects of TC pathfinding observed in vivo, including the rostrocaudal axis of TC projections [10]. Using this in vitro assay, we tested whether the mantle region of the rostral part of the VTel contains a chemoattractive cue for axons originating from the rostral thalamus by performing grafts of the mantle (postmitotic) region isolated from the rostral VTel (heterotopic graft) or the caudal VTel (homotopic graft) into the caudal VTel of a whole-mount telencephalon (see Figure 2A). In control experiments, homotopic grafts (caudal VTel into the caudal VTel) axons originating from the rostral DTh (DTR; see Figure S11 for isolation) specifically invade the rostral domain of the VTel (arrow in Figure 2B) as observed in control nongrafted whole-mount telecenphalic cocultures (see also [10,11]). Therefore, grafting itself does not perturb the topography of DTR axon projections in the VTel. However, when a small explant of rostral VTel is grafted heterotopically into the caudal VTel, rostral thalamic axons are significantly attracted towards the caudal VTel (arrows in Figure 2C; see also quantification in Figure 2D), overall randomizing the outgrowth of rostral DTh axons in the VTel. This result strongly suggests the presence of a chemoattractive cue for rostral thalamic axons in the rostral part of the VTel.

**Figure 5:**
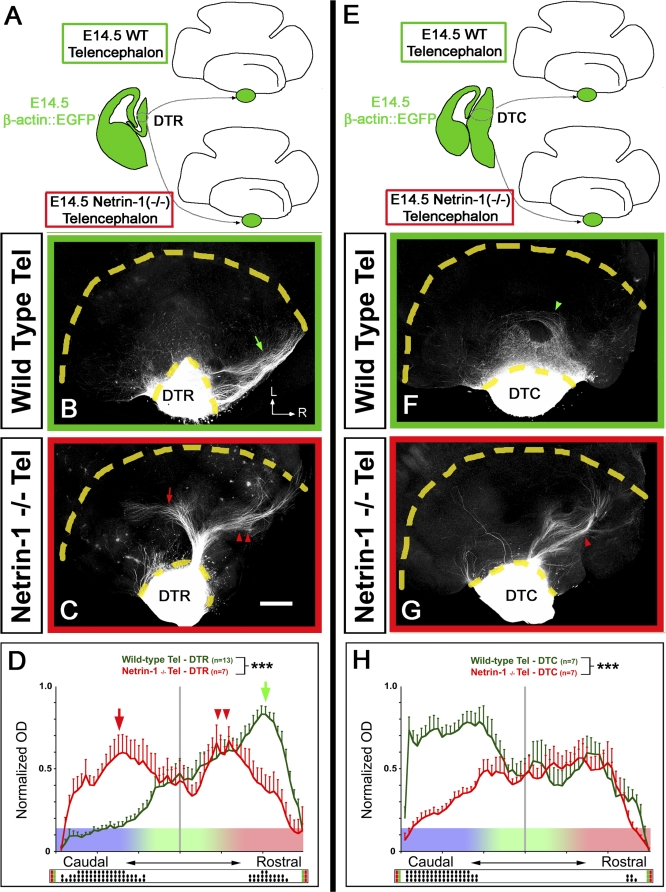
Netrin-1 Is Required in the Ventral Telencephalon to Specify the Topography of Projection of Both Rostral and Caudal Thalamic Axons (A and E) Schema of the experimental paradigm: rostral (DTR; [A–D]) or caudal (DTC; [E–H]) thalamic explants isolated from β-actin::EGFP E14.5 embryos were cocultured with E14.5 whole-mount telencephalon from either wild-type isochronic embryos (B and F) or *Netrin-1* knockout isochronic embryos (C and G) in order to test for the requirement of Netrin-1 specifically in the VTel. (B and C) DTR axons show a strong preferential outgrowth in the rostral part of the wild-type VTel (green arrow in [B]). A contingent of wild-type DTR axons grow significantly more caudally in a Netrin-1–deficient VTel (red arrow in [C]), whereas another contingent of TC axons maintains its projection to the rostral part of the VTel (double arrowheads in [C]). L, lateral; R, rostral. (D) Quantification of normalized optical density (OD) of DTR-EGFP axons growing in wild-type (*n* = 13, green) or *Netrin-1* knockout (*n* = 7, red) telencephalon. Significantly more EGFP-positive axons are growing in the caudal part of the VTel in the *Netrin-1* knockout than in the wild-type telencephalon. Triple asterisks (***) indicate *p* < 0.001, ANOVA one-way test (bins vs. genotype). (F and G) Axons originating from the caudal DTh preferentially grow in the caudal part of wild-type VTel (green arrow in [F]) but grow significantly more rostrally in the Netrin-1–deficient VTel (red arrowhead in [G]). (H) Quantification of normalized optical density (OD) of DTC-EGFP axons growing in wild-type (*n* = 13, green) or *Netrin-1* knockout (*n* = 7, red) telencephalon. The raster-like dot plots presented under each histogram (D and H) represents the significance of individual bin comparisons between the two experimental conditions according to a Fisher PLSD post hoc test (a single dot [•] indicates *p* < 0.05; double dots [••] indicate *p* < 0.01; and triple dots [•••] indicate *p* < 0.001). Scale bars in (B, C, F, and G) represent 300 μm.

### Netrin-1 Is Expressed in a High-Rostral to Low-Caudal Gradient in the Ganglionic Eminence

**Figure 3:**
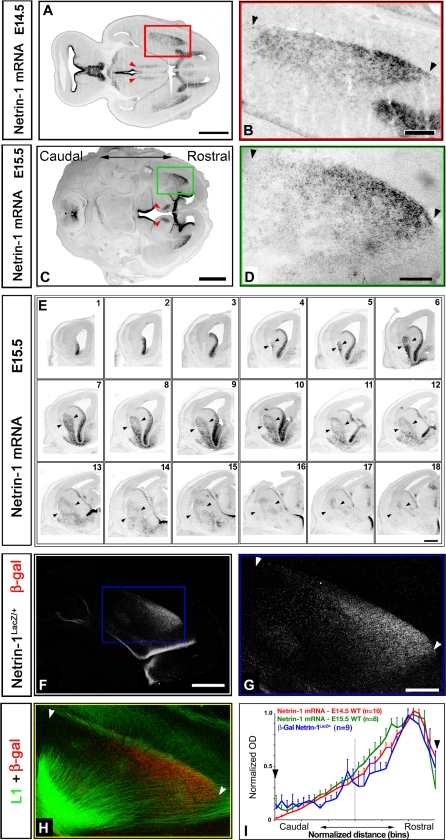
Netrin-1 Is Expressed in a High-Rostral to Low-Caudal Gradient in the Ganglionic Eminence (A–D) mRNA in situ hybridization performed on horizontal sections of E14.5 (A and B) and E15.5 (C and D) mouse embryos reveals that *Netrin-1* mRNA is expressed in a high-rostral to low-caudal gradient in the VTel. Also note that Netrin-1 is expressed in the DTh itself (arrowheads in [A] and [C]). (B) shows a higher magnification of the red boxed area in (A); (D) shows a higher magnification of the green boxed area in (C). (E) Series of 18 coronal sections from a single E15.5 wild-type mouse brain showing that the high-rostral to low-caudal gradient of *Netrin-1* mRNA expression is found in the mantle region of the ganglionic eminence. Sections are numbered from rostral (#1) to caudal (#18). Arrowheads indicate the location of the internal capsule. (F and G) This rostrocaudal gradient can also be visualized on horizontal sections of a *Netrin-1^LacZ/+^* E15.5 mouse embryo both at low (F) and high (G) magnification on horizontal sections immunostained for β-galactosidase. (H) This high-rostral to low-caudal gradient of Netrin-1 expression coincides spatially with TC axons in the internal capsule in the VTel as visualized by this double immunofluorescence for the cell adhesion molecule L1 (green) and β-galactosidase (red). (I) Quantification of the gradient of *Netrin-1* mRNA expression inside the VTel at E14.5 (red), E15.5 (green), and β-galactosidase immunofluorescence in a *Netrin-1^LacZ/+^* E15.5 mouse (blue) using normalized optical density measurement in 30 vertical bins oriented along the rostrocaudal axis on horizontal sections. *n* indicates the number of sections used to measure the normalized optical density values along the rostrocaudal axis. In (B, D, and G), arrowheads point to the rostrocaudal and dorsoventral width of the internal capsule within the VTel. Scale bars represent in (A) 800 μm; (B) 250 μm; (C) 1 mm; (D) 250 μm; (E) 80 μm; (F) 600 μm; and (G and H) 200 μm.

Earlier studies have shown that Netrin-1 is expressed in the mantle (postmitotic) region of the VTel of mouse embryos where the internal capsule forms [21–24]. We carefully examined the spatial pattern of Netrin-1 expression using two independent approaches at E14.5 and E15.5, when the vast majority of TC axons pioneer the VTel to form the internal capsule en route to the cortex in the mouse embryo [17,24]. First, using in situ hybridization performed on horizontal and coronal sections of E14.5 (Figure 3A and 3B) or E15.5 mouse embryos (Figure 3C–3E and 3I), we found that *Netrin-1* mRNA is expressed in a high-rostral to low-caudal gradient in the mantle region of the VTel.

We took advantage of a gene trap mouse line in which a LacZ expression cassette was inserted into the first intron of the *Netrin-1* coding sequence (*Ntn1^Gt(pGT1.8TM)629Wcs^* allele, abbreviated *Ntn1^LacZ^*; [21]). As shown in Figure 3F and 3G, anti-β-galactosidase immunofluorescence in E15.5 *Ntn1^LacZ/+^* mouse embryos recapitulates faithfully the graded expression of *Netrin-1* mRNA at the same age (Figure 3C and 3D). In order to examine the spatial relationship between this gradient of Netrin-1 expression and TC axons in the internal capsule, we performed L1 immunofluorescent staining in combination with anti-β-galactosidase immunofluorescence in *Ntn1^Lacz/+^* embryos at E15.5 (Figure 3H). A quantitative analysis of both *Netrin-1* mRNA expression and anti-β-galactosidase immunofluorescence along the rostrocaudal axis of the VTel reveals an almost linear high-rostral to low-caudal gradient (Figure 3I). Therefore, this gradient of Netrin-1 expression represents a good candidate to exert a function in the control of the topography of TC axons along the rostrocaudal axis of the VTel.

### Netrin-1 Is Necessary for the Establishment of the Topography of Thalamocortical Projections in the Ganglionic Eminence.

Inspection of the internal capsule of wild-type or *Netrin-1* knockout embryos at E18.5 using anti-L1 staining (which labels both TC and callosal, but not corticothalamic, axons [25]) failed to reveal any major axon outgrowth defect (see Figure S4): horizontal sections of E17.5 *Netrin-1* knockout embryos revealed no obvious decrease in the number of thalamic axons compared to wild-type littermates at the level of (1) the thalamic peduncle (axon bundle crossing the diencephalic to telencephalic boundary), (2) the internal capsule, or (3) the corticostriatal boundary compared to wild-type embryos (Figure S4). This qualitative analysis suggested that Netrin-1 is not simply required in vivo for proper outgrowth of thalamic axons into the internal capsule as suggested previously [22]. As shown later using whole-mount telencephalic cocultures, wild-type DTR or caudal DTh (DTC) axons growing in the VTel of Netrin-1–deficient embryos confirms quantitatively the absence of axon outgrowth defect compared to control wild-type telencephalon. Therefore, we conclude that Netrin-1 expression is not required for extension of thalamic axons in the VTel.

In order to test whether Netrin-1 controls the *guidance* of TC projections in the VTel, we performed BDA microinjections in the DTh of both wild-type (*n* = 34) and *Netrin-1^LacZ/LacZ^* (*Netrin-1^−/−^*; *n* = 17) E18.5 embryos. A qualitative illustration of the type of topographic projection defect observed in the *Netrin-1* knockout is shown in Figure 4 A–4G following a relatively large injection of BDA (more than 5% of DTh volume; injection not used for our quantitative analysis) in the rostral part of the DTh of a control (*Netrin-1^+/−^*; Figure 4B–4D) or *Netrin-1* knockout embryo (Figure 4E–4G). Using an oblique plane of section revealing the entire tract of TC projections from the DTh to the cortex (see Figure 4A; [26]), we show that thalamic axons originating from the rostral DTh invade more-caudal territories of the VTel of *Netrin-1* knockout embryos (arrows in Figure 4E–4G) than in control embryos (Figure 4B–4D).

Our quantitative analysis of a large number of BDA injections in E18.5 embryos reveals a profound disruption of the topography of TC projections in the *Netrin-1* knockout mouse (see Figures 4 and S5). The significance of the differences between each axon density map of TC projections is tested statistically using a two-way analysis of variance (ANOVA) test comparing *Netrin-1^+/+^* and *Netrin-1^−/−^* embryos (Figure S6). First, when clustered along the rostrocaudal axis of the DTh, the most significant differences in the pattern of TC projections concerns axons originating from the rostral thalamus, which reach the dorsal telencephalon at significantly more-caudoventral levels of the CSB. Thalamic axons originating from both the medial and caudal third of the DTh reach the CSB at a significantly more-rostral level in the *Netrin-1^−/−^* embryos than in wild-type control (Figures 4J, 4K, and S6B–S6D)

Similar disruption of the topography of TC projections in the VTel is visible when examining the mediolateral organization of thalamic projections (Figure 4L–4O): axons originating from the medial and central part of the DTh reach the CSB at more-caudoventral levels in *Netrin-1* knockout compared to wild-type mouse embryos. Additionally, axons originating from the lateral-most third of the DTh in *Netrin-1* knockout embryos are significantly shifted rostrodorsally at the level of the CSB compared to wild-type control (Figure 4O and S6H).

Using a reciprocal analysis, we confirmed these results by categorizing neuron position within the DTh based on the rostrocaudal level at which their axons cross the CSB (Figure 4P). This analysis confirms the severe disruption of the topography of thalamic projections characterizing the *Netrin-1* knockout embryos. Axons crossing the CSB at its rostral-most third originate from the rostromedial part of the DTh in wild-type embryos (green in Figures 4Q and S6J), but in contrast, originate from a more widespread area of the DTh in the *Netrin-1* knockout, including the extreme caudolateral territories of the DTh (Figures 4Q and S6J). Axons crossing the CSB along its medial third originate from a more-caudolateral domain of the DTh in *Netrin-1* knockout compared to wild-type littermates (Figures 4R and S6K). Strikingly, the reverse is found for thalamic axons crossing the CSB along its caudal third, where cell bodies are found in a more-rostromedial position of the DTh in *Netrin-1* knockout than in wild-type littermates (Figures 4S and S6L).

We also examined whether Netrin-1 is controlling the segregation of thalamic axons along the dorsoventral axis of the VTel. Using the reverse anatomy approach, we found that in wild-type mice, thalamic axons crossing the dorsal half of the CSB tend to originate from more-rostral domains of the DTh, whereas thalamic axons crossing the ventral half of the CSB tend to originate from the caudal DTh (green in Figure S7). The segregation along this axis is less marked than along the rostromedial to caudolateral axis (see Figure 1). We found small but significant differences in thalamic axon segregation along the dorsoventral axis of the CSB between control and Netrin-1–deficient embryos (Figure S7) suggesting that Netrin-1 might play a role in segregating thalamic axons along the dorso-ventral axis of the internal capsule.

### Graded Netrin-1 Expression in the Ventral Telencephalon Is Required for Proper Topographic Sorting of Thalamocortical Projections

These results are surprising because they suggest that Netrin-1 gradient in the VTel is not only attracting rostromedial thalamic axons in the rostral part of the VTel, but might also act as a repulsive cue for caudolateral thalamic axons. In other words, in the absence of Netrin-1 in vivo, rostromedial thalamic axons are shifted caudally according to their responsiveness to Netrin-1 (compatible with the removal of a rostral attractant in the VTel), but at the same time, caudolateral thalamic axons are shifted rostrally according to their responsiveness to Netrin-1 (compatible with the removal of a rostral repulsive cue in the VTel).

However, there is a potential caveat with this interpretation: Netrin-1 is not only expressed in the VTel, it is also expressed in the DTh itself (see arrowheads in Figure 3A and 3C). Therefore, at this point, we could not exclude that some of the topographic defects of TC axon outgrowth observed in vivo in the *Netrin-1* knockout embryos could be due to Netrin-1 expression in the DTh itself.

In order to test whether the graded expression of Netrin-1 in the VTel is required for the establishment of the topography of TC projections, we took advantage of our whole-mount telencephalic coculture assay in order to uncouple the genotype of the DTh and the telencephalon (see [10,11]). As shown in Figure 5A and 5E, we performed whole-mount cocultures between wild-type E14.5 EGFP-expressing dorsal thalamic explants (rostral DTh, Figure 5A–5D; or caudal DTh, Figure 5E–5H) with telencephalic vesicles isolated from isochronic wild-type (Figure 5B and 5F) or *Netrin-1* ^−/−^ embryos (Figure 5C and 5G).

Our results show that in the absence of Netrin-1 in the VTel, a significant proportion of axons originating from the rostral part of the DT are shifted caudally (red arrow in Figure 5C) compared to control cocultures (arrow in Figure 5B). However, a contingent of rostral thalamic axons is still projecting to the rostral third of the VTel (arrowheads in Figure 5C). The quantification of these cocultures (Figure 5D) demonstrates that axons originating from the rostral DTh and growing in Netrin-1–deficient telencephalon can be subdivided into two subpopulations that are both significantly shifted caudally (two peaks in Figure 5D) compared to control cocultures (green arrow in Figure 5D).

Next, we performed whole-mount telencephalic cocultures using axons originating from the caudal part of the DTh (DTC). As shown previously [10], DTC axons diffusely invade caudal territories of the VTel (Figure 5F and 5H). However, caudal DTh axons growing in a Netrin-1–deficient telencephalon do not show a preferential caudal outgrowth (arrowhead in Figure 5G) but instead display a more random distribution in the VTel (Figure 5H; see also Figure S8). Taken together, these results demonstrate that the high-rostral to low-caudal gradient of Netrin-1 expression in the VTel is required for the differential topographic mapping of thalamic axons before they reach the cortex. These results also suggest that the function of Netrin-1 in the topographic sorting of TC axons in the VTel requires both its attractive and repulsive properties.

### Netrin-1 Is Attractive for Rostral Thalamic Axons and Repulsive for Caudal Thalamic Axons

We first tested whether Netrin-1 differentially affects DTh axon populations through either attractive or repulsive activity, by testing directly whether Netrin-1 has a differential effect on different TC axons using collagen cocultures between E14.5 DTR or DTC explants and aggregates of HEK 293 that are stably expressing Netrin-1 [27] (Figure S9). These results show that DTR axons are significantly attracted by Netrin-1 (in accordance with [22,28]), but at the same time, that DTC axons are not attracted, but rather moderately repulsed, by Netrin-1 in vitro. One of the drawbacks of this collagen coculture assay is that TC axons are not growing through their “natural” environment, and therefore, axon responsiveness to specific axon guidance cue could be biased because axons do not express the right complement of axon guidance receptors. A precedent for this has been well documented in the developing spinal cord where commissural axons only up-regulate surface expression of Robo receptors after crossing the midline and therefore are not responding to the midline repellent Slits before they reach the midline [29].

**Figure 6:**
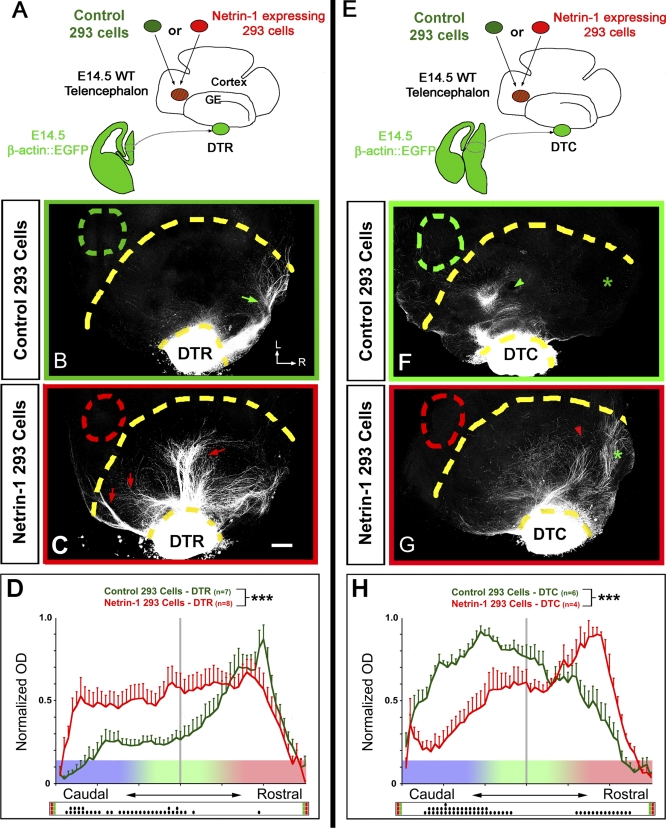
Netrin-1 Is Acting as a Chemoattractive Cue for Rostral Thalamic Axons and a Chemorepulsive Cue for Caudal Thalamic Axons (A and E) Experimental paradigm: control 293 cells (B and F) or 293 cells stably expressing Netrin-1 and embedded in collagen (C and G) were grafted in proximity of the caudal part of the VTel of E14.5 wild-type whole-mount telencephalon cocultured with EGFP-expressing explants isolated from isochronic rostral DTh (DTR; [B and C]) or caudal DTh (DTC; [F and G]). (B and C) Axons originating from the rostral DTh grow rostrally in the VTel of telencephalic whole mount grafted with control 293 cells in the caudal VTel (green arrow in [B]). In contrast, a significant proportion of DTR axons project caudally when Netrin-1–expressing cells are grafted in the caudal VTel (red arrows in [C]). L, lateral; R, rostral. (D) Quantification of normalized optical density (OD) of DTR-EGFP axons growing in VTel grafted caudally with control 293 cells (green) or VTel grafted caudally with Netrin-1–expressing 293 cells (red). (F and G) Axons originating from the caudal DTh grow caudally in the VTel of a telencephalic whole mount with control 293 cells grafted in the caudal VTel (green arrowheads in [F]). In contrast, a significant proportion of DTC axons grow rostrally when Netrin-1–expressing cells are grafted in the caudal VTel (red arrowhead in [G]). (H) Quantification of normalized optical density (OD) of DTC-EGFP axons growing in VTel with control 293 cells grafted caudally (green) or VTel with Netrin-1–expressing 293 cells grafted caudally (red). Significantly more DTC axons grow to the rostral part of the VTel grafted with Netrin-1–expressing cells than in control graft. Triple asterisks (***) indicate *p* < 0.001, ANOVA one-way test (overall effect: bins versus experimental conditions). The raster-like dot plot presented under each histogram (D and H) represents the significance of individual bins comparisons performed between the two experimental conditions according to a Fisher PLSD post hoc test (a single dot [•] indicates *p* < 0.05; double dots [••] indicate *p* < 0.01; and triple dots [•••] indicate *p* < 0.001). Scale bars in (B, C, F, and G) represent 150 μm.

In order to better test the differential effects of Netrin-1 on the response of thalamic axons originating from the rostral and caudal thalamus in a contextual environment, we performed whole-mount telencephalic assays in which a source of Netrin-1–expressing HEK293 cells ([23]) is ectopically grafted in the caudal VTel (see Figure 6A and 6E). Axons originating from the rostral thalamus now invade more-caudal territories of the VTel, overriding the repulsive effect of ephrin-A5 and other putative caudal repulsive activity [11], suggesting that they are attracted towards a caudal source of Netrin-1 (arrows in Figure 6C), whereas the control 293 cells graft has no effect (Figure 6B). Interestingly, the reverse is found for caudal thalamic axons, which are significantly shifted rostrally when confronted with a caudal source of Netrin-1 (Figure 6G and 6H) as compared to control grafts (Figure 6F and 6H). Note that this caudal source of Netrin-1 imposed experimentally is likely to disrupt the endogenous gradient of Netrin-1 still present at high levels in the rostral part of the VTel (see Figure 3). These results strongly suggest that in the VTel, Netrin-1 functions as a chemorepulsive cue for caudal thalamic axons and a chemoattractive cue for rostral thalamic axons.

### Netrin-1 Receptors Are Expressed in Complementary Domains in the Dorsal Thalamus

**Figure 7:**
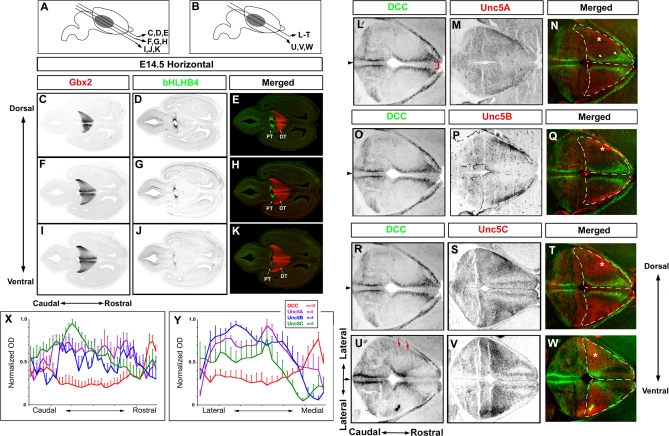
Patterns of Expression of Netrin-1 Receptors in the Mouse Dorsal Thalamus (A and C–K) At E14.5, mRNA in situ hybridization (ISH) for *Gbx2* delineates the DTh along its rostrocaudal axis on horizontal sections distributed along the doesoventral axis of the DTh ([C, F, and I]; see levels of section in [A]). Expression of *bHLHB4* on adjacent sections delineates the pretectum (PT) (D, G, and J). When merged (E, F, and K), *Gbx2* and *bHLHB4* show nonoverlapping and complementary expression at the diencephalic–mesencephalic boundary delineating the caudal limit of the DTh (DT) on E14.5 horizontal sections. (B and L–W) In situ hybridization for *DCC* ([L, O, R, and U], green in [N, Q, T, and W]), *Unc5A* ([M], red in [N]), *Unc5B* ([P], red in [Q]), and *Unc5C* ([S and V], red in [T and W]) on adjacent horizontal sections of E14.5 mouse embryos isolated at two levels of sections along the dorsoventral axis of the DTh (B). *DCC* is expressed most highly in a rostromedial domain of the DTh in postmitotic regions (unpublished data) and is excluded from the thin ventricular zone left at this time (bracket in [L]). Conversely, *Unc5A*, *Unc5B*, and *Unc5C* are all expressed in nonoverlapping caudolateral domains of the DTh (stars in [N, Q, T, and W] indicate the approximate peak of expression). The dashed lines in (N, Q, T, and W) correspond to the actual limit of the DTh as defined by *Gbx2* expression on adjacent sections (see [I–K]). Note that *DCC* is expressed at low, but significant, levels in the caudolateral domain of the DTh (red arrows in [U]) where it is coexpressed with *Unc5C* (star in [V]). (X and Y) Quantification of the gradient of *DCC* (*n* = 10), *Unc5A* (*n* = 5), *Unc5B* (*n* = 4), and *Unc5C* (*n* = 6 sections) mRNA expression along the rostrocaudal axis (X) and the mediolateral axis (Y) of the DTh at E14.5 as indicated by the lines in (L). Gradients were measured by normalizing the optical density values on multiple adjacent sections (number indicated in [Y]) shown in (L–W). Arrowheads in (L, O, R, and U) indicate the midline.

So far, our results imply that dorsal thalamic neurons express different Netrin-1 receptors conferring attractive (for DTR axons) or repulsive (for DTC axons) responses to Netrin-1. In order to substantiate this hypothesis, we examined the pattern of expression of several transmembrane receptors known to mediate Netrin-1 responsiveness: Deleted in Colorectal Cancer (DCC), known to mediate chemoattraction to Netrin-1 [27], and homologs of the Caenorhabditis elegans Unc5 receptor called Unc5A–C (also called Unc5H1–3), known to mediate chemorepulsion to Netrin-1 upon heterodimerization with DCC [30,31]. A fourth mammalian ortholog of *Unc5* has been recently identified, but its affinity for Netrin-1 has not been assessed yet [32]. We performed in situ hybridization for *DCC* and *Unc5A*, *Unc5B* and *Unc5C* (Figure 7) on serial horizontal sections of E14.5 mouse embryos in order to best visualize differences of Netrin-1 receptor expression along the rostromedial to caudolateral axis of the DTh.

First, we wanted to define accurately the caudal extent of the DTh on horizontal sections from E14.5 mouse embryos. To do this, we used two markers: first the transcription factor *Gbx2*, which is a reliable marker of the DTh at E14.5 [33,34], and the transcription factor *bHLHB4*, which has been recently identified as a marker of the pretectum, which is immediately caudal to the DTh during embryogenesis [35]. Our results show that these two markers reliably identify the caudal limit of the DTh on horizontal sections of E14.5 mouse embryos along the dorsoventral axis of the diencephalic–mesencephalic boundary (Figure 7C–7K). Therefore, in the rest of our analysis, we used the caudal limit of *Gbx2* expression as a marker of the caudal limit of the DTh (see lines in Figure 7N, 7Q, 7T, and 7W).

We found that *DCC* mRNA is expressed at high levels in the rostromedial part of the DTh (Figures 7L–7Q and S10A and S10B) but is also expressed at lower levels in more-caudolateral territories of the DTh (arrows in Figures 7U and S10C). In contrast, *Unc5A*, *Unc5B*, and *Unc5C* are expressed in nonoverlapping caudolateral domains of the DTh (Figure 7M, 7N, 7P, 7Q, 7S, and 7T, respectively, and Figure S10D–S10L). The star in Figure 7N, 7Q, 7T, and 7W marks the peak of *Unc5A–C* expression. These complementary patterns of expression are compatible with our model, suggesting that axons originating from the rostromedial DTh (which are attracted by Netrin-1 in the rostral part of the VTel) express *DCC* only, whereas thalamic axons originating from the caudolateral domain of the DTh are repulsed by Netrin-1 in the rostral VTel and express *Unc5A–C*, as well as low levels of *DCC*. Interestingly, *DCC* and *Unc5A–C* are highly expressed in other parts of the diencephalon, including the epithalamus (Figure S10B, S10C, S10E, and S10H), the ventral thalamus (Figure S10B, S10E, and S10K), as well as in the pretectum (Figure 7L–7W), suggesting other functions during diencephalic/mesencephalic development.

### Blocking DCC Function Impairs the Ability of Rostral Dorsal Thalamus Axons to Grow Rostrally in the Ventral Telencephalon

**Figure 8:**
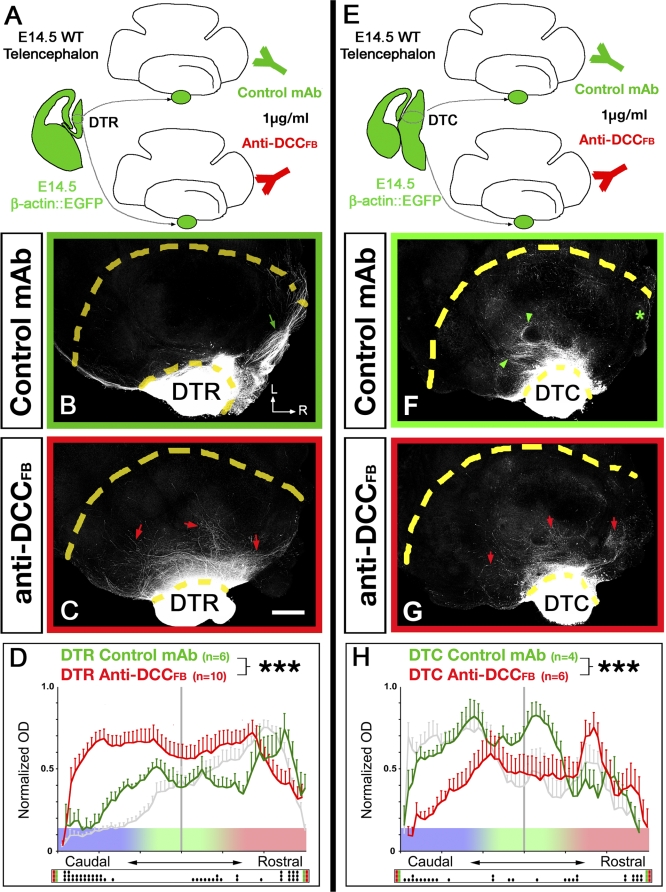
DCC Is Required for Both Attraction of Rostral Thalamic Axons and Repulsion of Caudal Thalamic Axons from the Netrin-1–Rich Rostral Domain of the Ventral Telencephalon (A and E) Isochronic whole-mount telencephalic cocultures with EGFP-expressing rostral (B–D) or caudal (F–H) DTh explants were incubated either with control isotype mouse IgG (B and F) or function-blocking anti-DCC monoclonal antibody (mAb) (C and G). Blocking DCC receptor function randomizes the outgrowth of both rostral and caudal DTh axons. L, lateral; R, rostral. (M) Quantification of normalized optical density (OD) of DTR-EGFP axons (D) or DTC-EGFP axons (H) growing in VTel with function-blocking anti-DCC antibodies (red curves) or control mouse anti-IgG antibodies (green curves). Triple asterisks (***) indicate *p* < 0.001, ANOVA one-way test (overall effect: bins versus experimental conditions). For comparisons, the gray curves represent the distribution of control DTR axons (in [D]) and control DTC axons (in [H]) cultured without antibody as shown in Figure 5D and 5H. The raster-like dot plot presented under each histogram represents the significance of individual bin comparisons performed between the two experimental conditions using a Fisher PLSD post hoc test (a single dot [•] indicates *p* < 0.05; double dots [••] indicate *p* < 0.01; and triple dots [•••] indicate *p* < 0.001).

We tested whether DCC is required in the topographic projection of thalamic axons by using a well-characterized function-blocking anti-DCC antibody (clone AF5 [27]) in the whole-mount telencephalic coculture assay (Figure 8A and 8E). Our results show that DTR axons specifically invade the rostral domain of the VTel when cultured in the presence of isotype-control mouse IgG (Figure 8B), but in the presence of function-blocking anti-DCC antibodies, DTR axon outgrowth is significantly randomized (Figure 8C) and grows significantly more caudally than in control cocultures (Figure 8D). Similarly, blocking DCC function tends to randomize the outgrowth of DTC axons, which invade significantly more-rostral domains of the VTel (arrow in Figure 8G and 8H) compared to DTC axons in control cocultures (Figure 8F and 8H). Overall, these results strongly suggest that DCC receptor function is required both for the attraction of DTR axons to rostral Netrin-1–rich territories of the VTel and for the repulsion of DTC axons away from the same domain.

### Unc5A/C Receptors Are Required for Caudal Dorsal Thalamus Axon Repulsion Away from the Rostral Domain of the Ventral Telencephalon

**Figure 9:**
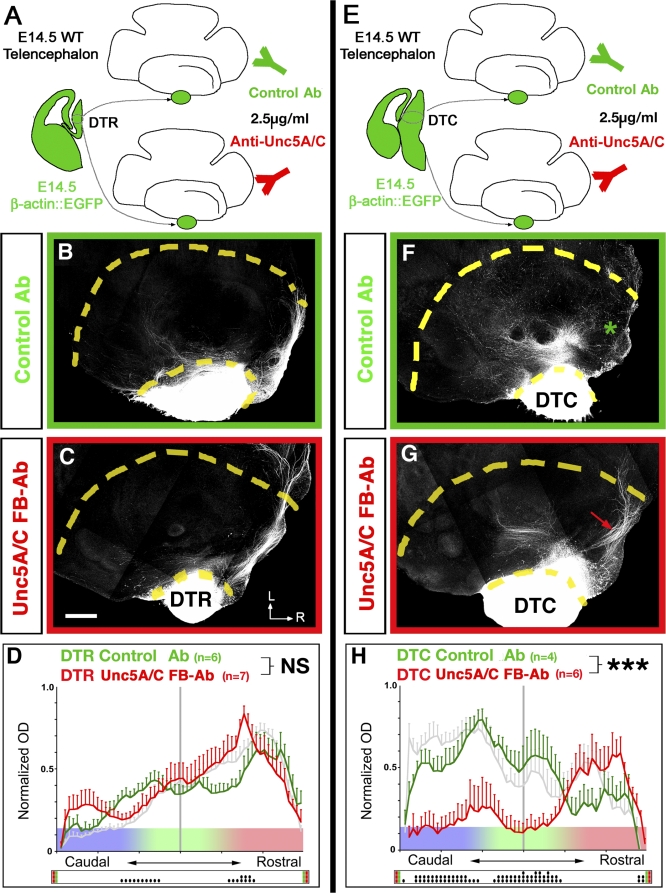
Unc5A and C Receptors Are Required for the Repulsion of Caudal DT Axons from the Netrin-1–Rich Rostral Domain of the Ventral Telencephalon (A and E) Isochronic whole-mount telencephalic cocultures with EGFP-expressing rostral (B–D) or caudal (F–H) DTh explants were incubated either with control isotype mouse IgG (B and F) or function-blocking anti-Unc5A/C polyclonal antibody (Ab) (C and G). Blocking the function of Unc5A/C receptors does not affect the rostral outgrowth of DTR axons (see [C and D]) but induces a significantly more-rostral outgrowth of DTC axons in the VTel (see [G and H]). Quantification of normalized optical density (OD) of DTR-EGFP axons (D) or DTC-EGFP axons (H) growing in VTel with function-blocking anti-Unc5A/C antibodies (red curves) or control mouse anti-IgG antibodies (green curves). NS, nonsignificant (*p* > 0.05); triple asterisks (***) indicate *p* < 0.001, ANOVA one-way test (overall effect: bins versus experimental conditions). For comparisons, the gray curves represent the distribution of control DTR axons (in [D]) and control DTC axons (in [H]) cultured without antibody as shown in Figure 5D and 5H. The raster-like dot plot presented under each histogram represents the significance of individual bin comparisons performed between the two experimental conditions using a PLSD-post-hoc test (a single dot [•] indicates *p* < 0.05; double dots [••] indicate *p* < 0.01; and triple dots [•••] indicate *p* < 0.001). L, lateral; R, rostral.

We next tested whether Unc5 receptor function is required for the topographic projections of DT axons in the VTel. We used a commercially available polyclonal antibody initially raised against the extracellular domain of Unc5H1 (anti-rat Unc5H1, R&D Systems) and reported to act as a function-blocking reagent against both Unc5A and Unc5C (Unc5H1 and Unc5H3, respectively [36]). We verified the cross-reactivity of this anti-rat Unc5H1 antibody with mouse Unc5A, 5B, and 5C proteins using a biochemical approach (see Figure S12). Our results show that anti-rat Unc5H1 binds to mouse Unc5A and Unc5C, but not Unc5B, and that its relative affinity for Unc5C when standardized to anti-myc immunoreactivity is about a third of its affinity for Unc5A (Figure S12). We used this reagent to block Unc5A/C receptor function in the whole-mount telencephalic assay using both DTR (Figure 9A–9D) and DTC (Figure 9E–9H). Our results show that blocking Unc5A/C receptor function does not have any significant effect on the guided outgrowth of DTR axons in the rostral domain of the VTel (Figure 9C and 9D) compared to control (Figure 9B and 9D). In contrast, blocking Unc5A/C receptor function had a highly significant effect on DTC outgrowth, inducing a significant shift of DTC axon outgrowth into the rostral Netrin-1–rich domain of the VTel (Figure 9G and 9H) compared to control (Figure 9F–9H). These results suggest that Unc5A/C receptors are required for the repulsion of DTC axons away from the rostral Netrin-1–rich domain of the VTel but do not play any role in the attraction of DTR axons towards the same region.

### Unc5 Receptor Overexpression in Rostral Thalamic Neurons Is Sufficient to Induce Caudal Outgrowth of Their Axons into the Ventral Telencephalon

**Figure 10:**
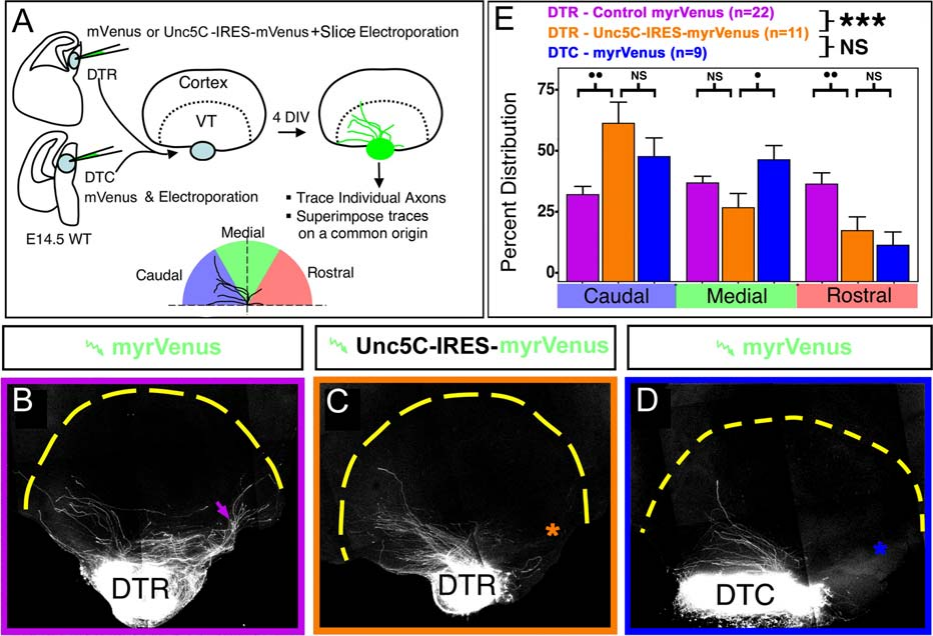
Expression of Unc5C in the Rostral Part of the Dorsal Thalamus Is Sufficient to Induce Repulsion of DTR Axons from Netrin-1–Rich Rostral Domain of the Ventral Telencephalon (A) Experimental approach: E14.5 250-μm–thick slices containing the rostral or caudal domain of the DTh were microinjected and electroporated using a control IRES-myristoylated (m)Venus or a Unc5C-IRES-mVenus expression plasmid. Immediately following electroporation, DTR or DTC explants were isolated and cocultured with a whole-mount telencephalon for 4 d in vitro (DIV). Following fixation and staining with anti-EGFP antibodies, individual DTR or DTC fluorescent axons or fascicles were traced and superimposed on a referenced-plot that was then quantified using ImageJ for optical density distribution in three radial bins. DIV, days in vitro; WT, wild type. (B–D) DTR (B) and DTC (D) axons electroporated with control mVenus-expression plasmid grow preferentially to the rostral and caudal domain of the VTel, respectively. However, DTR axons overexpressing Unc5C grow significantly more caudally than control DTR axons (B) in the VTel, suggesting that Unc5C expression is sufficient to convert DTR into the DTC pattern of axon growth in the VTel. (E) Quantification of the results shown in (B–D) analyzing the percentage of fluorescent axons located in caudal, medial, and rostral bins of the VTel Triple asterisks (***) indicate *p* < 0.001, ANOVA one-way test (overall effect: bins versus experimental conditions). NS, nonsignificant (*p* > 0.05); a single dot [•] indicates *p* < 0.05; and double dots [••] indicate *p* < 0.01: significance of individual bin comparisons performed between the two experimental conditions using a Fisher PLSD post hoc test.

We tested whether Unc5 receptor expression is the critical determinant of the difference between DTR and DTC axons towards Netrin-1 in the VTel. To do this, we overexpressed the Unc5C receptor in rostral thalamic neurons where it is normally expressed at low levels. We implemented an ex vivo slice electroporation technique developed recently by Cobos et al. [37]. Following focal microinjection of plasmid expressing myristoylated-(m)Venus or Unc5C-IRES-mVenus in the DTh and slice electroporation, explants corresponding to DTR or DTC were cocultured for 4 d in vitro with isochronic whole-mount telencephalon (Figure 10A). This technique results in clear visualization of single thalamic axons or small axon fascicles that were traced individually in ImageJ and plotted on a common reference for quantification (Figure 10A). Our results show that overexpression of Unc5C (but also Unc5A or B; unpublished data) is sufficient to convert the preferential outgrowth of DTR axons in the rostral domain of the VTel (Figure 10B) into outgrowth in the caudal domain of the VTel (Figure 10C) as observed with DTC axons (Figure 10D). The quantification (Figure 10E) demonstrates that the topography of DTR axon outgrowth overexpressing Unc5C does not differ from DTC axons but is significantly different from control DTR axons in the VTel. These results show that differential Unc5 receptor expression is a critical determinant in the topographic outgrowth of thalamic axons originating from the rostromedial compared to the caudolateral part of the thalamus in response to Netrin-1 in the VTel.

## Discussion

Our results provide novel insights into the molecular mechanisms patterning the topography of TC projections to specific cortical domains by controlling their guidance at the level of their main intermediate target, the VTel. We show that Netrin-1 is expressed in a high-rostral to low-caudal gradient in the VTel and demonstrate that the graded expression of Netrin-1 in the VTel is required cell nonautonomously for (1) attracting rostral thalamic axons in a DCC-dependent manner and (2) repulsing caudal thalamic axons in a DCC–Unc5 receptor-dependent manner. Our results show that the long-range gradient of Netrin-1 expression in the VTel confers a novel function to this well-characterized axon guidance cue: controlling the topographic mapping of large ensembles of axons, a function largely attributed to the ephrin–Eph signaling system and more recently to the Wnt/Ryk signaling [38].

### The Topography of Thalamocortical Projections Is Initiated in the Ventral Telencephalon

Recent studies provided evidence showing that TC axons are topographically organized in response to axon guidance cues located in the VTel [6,10,11]. However, the exact 3-D organization of TC axons in VTel, where they form the internal capsule with descending corticofugal axons, has remained elusive because of the lack of quantitative analysis. Qualitative analysis based on carbocyanine injections in single brains suggested that TC axons are segregated according to their origin in the DTh along the mediolateral axis [18,19,39] as well as the rostrocaudal axis [10]. Our quantitative analysis demonstrates that both axes are equally important, and we show that at the level of the CSB, i.e., before invading the cortex, thalamic projections are highly organized along a rostromedial to caudolateral axis (Figure 1). Therefore, there is a precise “blueprint” of the topography of TC projections generated before entering the cortex as suggested previously [5].

**Figure 11:**
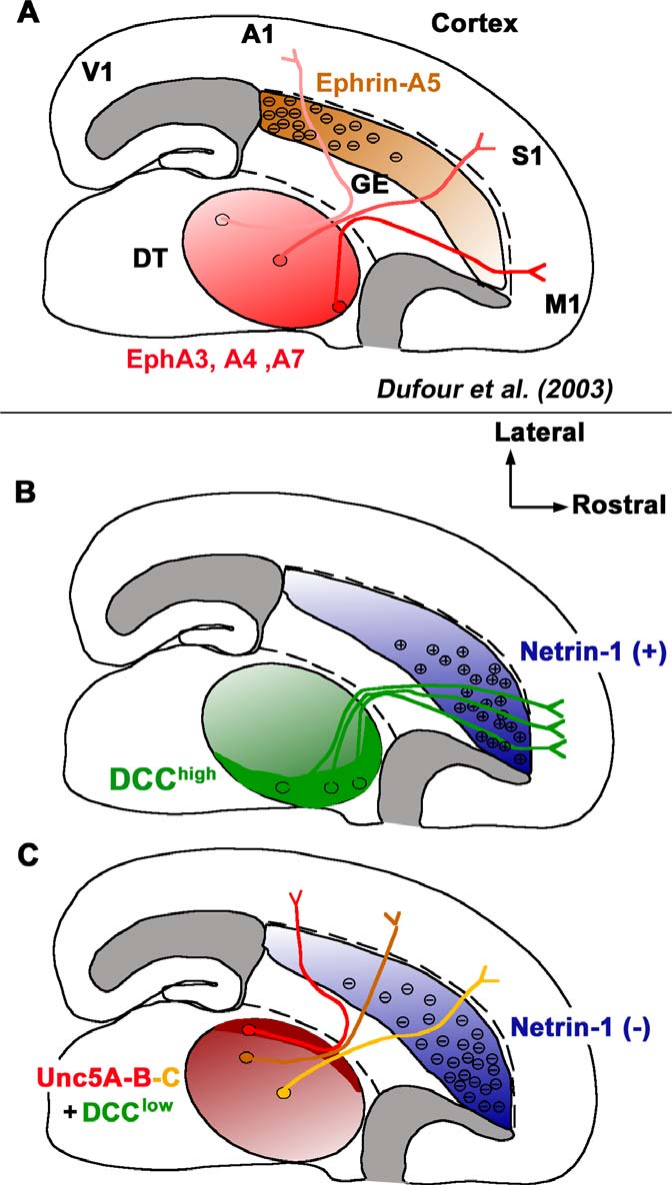
Model of the Role of Netrin-1 Signaling in the Topography of Thalamocortical Projections in the Ventral Telencephalon Schemas summarizing previous [11] and present findings regarding the axon guidance cues controlling the topographic sorting of TC axons in the VTel. (A) High rostromedial and low caudolateral gradient of EphA receptors (EphA3-4-7) mediate chemopulsion of rostromedial DTh (DT) axons to the high-caudal to low-rostral gradient of ephrin-A5 in the VTel. (B and C). In the present study, we demonstrate that a high-rostral to low-caudal gradient of Netrin-1 in the VTel plays a critical role in the topographic projection of DTh axons in the VTel. We show that the rostromedial domain of the DTh expresses high levels of DCC and that the caudolateral domain of the DTh expresses low levels of DCC, which is required both for the attraction of DTR axons and the repulsion of DTC axons to the Netrin-1–rich rostral domain of the VTel. We also show that Unc5A and B are expressed preferentially in the caudolateral domain of the DT, and Unc5C is expressed in a high-caudolateral to low-rostromedial gradient in the DTh. Finally, we provide evidence that (1) Unc5A/C are required for the repulsion of caudal DTh axons from the Netrin-1–rich domain of the VTel, but (2) they do not play any significant role in the projection of rostromedial DTh axons, and (3) that overexpression of Unc5C in DTR axons is sufficient to convert their outgrowth into DTC outgrowth, i.e., repulsion away from the Netrin-1–rich rostral domain of the VTel. A1, primary auditory area; GE, ganglionic eminence; M1, primary motor area; S1, primary somatosensory area; V1, primary visual area.

Where exactly is this topography initiated within the VTel? Axons entering the VTel show a loose degree of organization when pioneering the internal capsule, and axons originating from different regions of the thalamus have to redistribute or “fan out” over a large area: at E14/15, thalamic axons pioneer the internal capsule as a bundle referred to as the thalamic peduncle, roughly 100–200-μm wide along its rostrocaudal axis. These axons will redistribute over approximately 2–3 mm when they reach the CSB and enter the cortex. Based on previous and present results, we proposed that TC axon sorting occurs progressively as the axons grow along the mediolateral axis of the VTel [5] (Figure 11). Interestingly, the only two axon guidance molecules (ephrin-A5; [11] and Netrin-1; present study) identified so far as playing a significant role in this topographic sorting of TC axons in the VTel are both expressed in the most lateral part of the mantle region of the VTel and are therefore likely expressed by postmitotic neurons forming the striatum. Future studies will address how opposing gradients of ephrin-A5 and Netrin-1 are generated. Two interesting possibilities come to mind: first, this gradient is the result of patterning cues such as Shh or fibroblast growth factors (FGFs) specifying the rostrocaudal identity of ventral telencephalic regions, and/or second, this graded expression of Netrin-1 is the result of a graded density of cells migrating rostrocaudally within the VTel from a point source.

### Critical Role of Netrin-1 in the Establishment of TC Projections in the Ventral Telencephalon

Recent evidence suggests that thalamic axons' responsiveness to Netrin-1 expressed in the VTel is modulated by extracellular serotonin levels [39]. Bonnin et al. [39] reports that in vitro, in the absence of serotonin, Netrin-1 is repulsive to “anterior” thalamic axons and attractive to “posterior” thalamic axons, which seems at odds with our present results. However, the authors provide evidence that in the presence of high concentrations of serotonin (30 μM), these responses are reversed, and now Netrin-1 is attractive to anterior thalamic axons and repulsive to posterior thalamic axons. Several technical differences might account for the potential discrepancies between these and our results. First, Bonnin et al. are microdissecting very small parts of the DTh “by hand,” whereas, as previously published by our group (see also Figure S11), we can only isolate thalamic explants reproducibly from serial 250-μm–thick vibratome slices [10]. Second, as previously shown, serotonin levels are high in rodent embryonic tissue; since serotonin is present at high levels in the maternal circulation and undergoes specific uptake in the placenta [40,41], one would expect axons throughout the mouse embryo to be exposed to high levels of extracellular serotonin. We did test the responsiveness of DTR or DTC explants to a source of Netrin-1 in a collagen assay in the presence of horse serum-containing medium (presumably containing levels of serotonin comparable to blood levels found in vivo) and confirmed that DTR axons are attracted by Netrin-1 source but that DTC are either not responsive or slightly repulsed by a source of Netrin-1 in these in vitro conditions (Figure S9). More importantly, knockout mice for the main vesicular monoamine transporter (VMAT2) expressed in the embryonic brain have been generated and show undetectable levels of all three major monoamines (serotonin, dopamine, and noradrenalin) in the developing brain [42]. These mice show a delayed, but otherwise unaffected, TC development, and certainly no defect in the topography of ventrobasal (VB) thalamic axons targeting to the primary somatosensory cortex (S1) [42]. These and other data strongly suggest that serotonin and other monoamines are not required for the initial establishment of the topography of TC projections in vivo [42].

Regardless of the intracellular signaling pathways mediating Netrin-1 signaling in vivo, our quantitative analysis of axon tracing in wild-type and *Netrin-1* knockout embryos (Figures 4 and S5–S7) provides unequivocal genetic evidence for the requirement of Netrin-1 in vivo for (1) the preferential growth of rostromedial thalamic axons into the rostrodorsal part of the VTel and (2) for the preferential growth of caudolateral thalamic axons into the caudoventral part of the VTel. The fact that in the *Netrin-1* knockout embryos, axons originating from the rostromedial domains of the DTh are significantly shifted caudoventrally compared to controls and axons originating from the caudolateral domains of the DT are significantly shifted rostrodorsally (Figures 4 and S6) compared to control embryos (Figure 1) represents the strongest evidence in favor of our model suggesting that Netrin-1–rich rostral domain of the VTel normally acts as an attractant for rostromedial thalamic axons and a repulsive cue for caudolateral thalamic axons. This model is corroborated by our DCC and Unc5A–C expression data and more significantly by our function-blocking experiments demonstrating that a high level of DCC receptor expression by rostromedial thalamic axons mediates attraction towards the Netrin-1–rich domain of the VTel (Figure 8) and that both DCC and Unc5A–C expression are required for repulsion of axons originating from caudolateral domain of the DTh away from the Netrin-1–rich rostral domain of the VTel (Figure 9).

Interestingly, Netrin-1 is also required for the organization of thalamic axons along the dorsoventral axis of the developing DTh (see Figure S6), an axis that is not tightly associated with differences of expression of DCC and/or Unc5A–C. Future experiments will explore whether other Netrin-1 receptors such as Neogenin or the recently identified Unc5D (Unc5H4) are differentially expressed along the dorsoventral axis of the DTh and mediate segregation of thalamic axons in the VTel.

### Establishment of Topographic Maps: General Requirement for Several Counterbalancing Gradients?

In the retinotectal projection, axons located along the nasotemporal axis of the retina are topographically mapped along the anteroposterior axis of the optic tectum through the action of EphA–ephrinA signaling system [12], whereas axons originating along the dorsoventral axis of the retina project topographically along the mediolateral axis of the tectum. Recent evidence demonstrates that the topography established along the mediolateral axis of the optic tectum is regulated by EphB–ephrinB signaling (reviewed in [12,43]). However, theoretical modelization suggested that the graded expression of a single axon guidance cue is not sufficient for specifying a continuous topographic map along both axis of the tectum; at least one other gradient of an additional cue is necessary for proper topographic map formation in the retinotectal system [44–47]. Indeed, recent evidence shows that a gradient of Wnt3 along the mediolateral axis of the tectum counterbalances the attractive function of ephrinB1 [13].

Our results also suggest that a Netrin-1 gradient counterbalances the function of the ephrin-A5 gradient identified in the VTel [11] and that the combined expression of these two cues (possibly along with other graded cues) is required for the establishment of TC topography in the VTel (Figure 11). The main difference between the retinotectal and the TC projections is that TC axons are first sorted in the VTel, their main intermediate target, before they reach their final target, the cortex. This reflects the “nested” nature of TC projections: at embryonic stages, axons from distinct parts of the mouse thalamus (ultimately corresponding to different thalamic nuclei) are first sorted to different cortical domains in the intermediate target, the VTel (*interareal* topography), but at early postnatal stages, neurons in each sensory thalamic nuclei project topographically within each cortical area (intra-areal mapping; i.e.; sensory map formation) [48]. Interestingly, the sorting of axons along the rostromedial to caudolateral axis of the thalamus at the level of the internal capsule (i.e., before they reach the cortex) is perfectly conserved in humans as shown recently by tract-tracing studies using diffusion tensor imaging [49], which suggests that the establishment of TC topography in the VTel is the result of an evolutionary conserved developmental mechanisms in mammals.

### Regionalization of the Dorsal Thalamus and Specification of the Topography of Thalamocortical Projections

Importantly, the basic topography of TC projections is specified in the VTel before individual thalamic nuclei can be identified cytoarchitecturally [10,11,34]. Careful examination of the expression pattern of several transcription factors, including *Ngn2*, *Lhx2*, *Lhx9*, and *Gbx2*, from E12 to postpartum day 0 (P0) demonstrated that their expression is regionalized between E12 and E14, well before the appearance of distinct thalamic nuclei (E15–E16) [34]. These results suggested that phenotypic traits of thalamic neuron identity, such as their patterns of axon projections, are intrinsically specified by the combinatorial expression of transcription factors, a model based on specification of motor neuron identity in the developing spinal cord [50,51]. Interestingly, an experimental validation of this model was provided recently by the analysis of the function of the bHLH transcription factor Ngn2, which specifies the topography of TC projections to the frontal cortex by controlling the responsiveness of thalamic axons to so-far unidentified intermediate axon guidance cues present in the VTel [10]. Based on the present results, we can hypothesize that the caudal shift displayed by rostral thalamic axons of the Ngn2 knockout embryos in the VTel could be due to down-regulation of Netrin-1, or ephrin-A5 responsiveness. Further experiments will determine whether Netrin-1 and ephrin-A5 receptors examined in this and previous studies (*DCC*, *Unc5A–C*, and *EphA4*) have altered expression profiles in the DTh of *Ngn2* knockout embryos.

### Role of Netrin-1 as an Intermediate Axon Guidance Cue in the Ventral Telencephalon

Several studies have implicated Netrin-1 as an intermediate target cue for both corticofugal [23] and TC axons [22,39]. Netrin-1 was first shown to stimulate the outgrowth of descending corticofugal axons in vitro and attract these axons towards the internal capsule [23]. Interestingly, Netrin-1 expression in the VTel has also been proposed to stimulate thalamic axon outgrowth [22]. This study provided evidence for a decreased number of thalamic axons invading the VTel as well as a disorganized internal capsule, using DiI tracing and L1 staining. Despite careful examination, we did not find evidence of a significant decrease in the number of thalamic axons in the VTel of *Netrin-1* ^−/−^ embryos compared to control (see Figure S4), and the longest DTh axons' length was not significantly altered when axons were growing in *Netrin-1* ^−/−^ or control VTel (Figure S8). Furthermore, in our whole-mount telencephalic assay, wild-type DTR axons grew equally well in a wild-type or a Netrin-1–deficient VTel (Figure 5), suggesting that Netrin-1 does not play a critical role in the stimulation of thalamic axon outgrowth in vivo. The potential discrepancies between our results and the study by Braisted et al. (2000) [22] could be due to methodological differences or to differences in the genetic background of the *Netrin-1* knockout mice between the two studies. As a precedent, mice presenting a null mutation in the Netrin-1 receptor *Unc5C* on the inbred C57BL/6J (B6) genetic background display abnormal projections of both trochlear nerve and motor neuron axons, but these defects are greatly attenuated on a hybrid B6 × SJL background [52]. The authors have provided evidence for a locus representing a genetic suppressor of Unc5C function on mouse chromosome 17 [52].

### DCC and Unc5 Receptors Mediate the Differential Responsiveness to Netrin-1 in Rostral and Caudal Thalamic Axons

In mammals, there are at least five genes encoding transmembrane receptors for Netrin-1: DCC (Deleted in Colorectal Cancer) and Neogenin receptors mediate the attractive response elicited by Netrin-1, whereas Unc5A–C family members mediate repulsion elicited by Netrin-1 either as homodimers or heterodimers with DCC [53,54]. Our current results show an interesting regionalization of Netrin-1 receptor expression along the rostromedial to caudolateral axis of the DTh. At E14.5, when the topography of TC axons is initiated in the VTel, but before individual thalamic nuclei are formed, *DCC* is expressed at high levels in a rostromedial domain of the DTh, whereas *Unc5A*, *Unc5B*, and *Unc5C* are expressed in largely nonoverlapping caudolateral domains of the DTh (see Figure 7). Interestingly, *Unc5C* expression pattern is more widespread than Unc5A and B, and seems to overlap at least partially with the rostromedial domain of DCC expression (see Figure 7). Our function-blocking experiments demonstrate that DCC is required for the guidance of rostral thalamic axons to the Netrin-1–rich rostral domain of the VTel, whereas DCC and Unc5C are required for the proper repulsion of caudal thalamic axons to the same Netrin-1–rich region.

A recent study has implicated ephrin-A5–EphA4 signaling in the initiation of the topography of TC axon projection in the VTel [11]. Three EphA receptors (*EphA4*, *A3*, and *A7*) were shown to be expressed in high-rostromedial to low-caudolateral gradients in the E14.5 DTh, whereas the ephrin-A5 ligand was found to be expressed in a high-caudal to low-rostral gradient in the VTel (Figure 11A). This study also provided in vivo and in vitro functional evidence demonstrating that both ephrin-A5 expression in the VTel and EphA4 expression in the DTh were required for the proper topographic projection of thalamic axons [11]. Taken together, our results and those of Dufour et al. (2003) [11] suggest that rostromedial thalamic neurons express high levels of DCC and EphA receptors conferring to their axons both attractive responsiveness to rostral Netrin-1 and repulsive responsiveness to caudal ephrin-A5, respectively, resulting in repulsion from the caudal domain and attraction to the rostral domain of the VTel. Our DCC function-blocking experiments demonstrate that DCC is required for the attraction of rostral thalamic axons to the rostral domain of the VTel. In contrast, progressively more-caudolateral thalamic neurons express lower levels of EphA receptors and higher levels of Unc5A–C receptors, and we demonstrate that this decreased sensitivity to the repulsive effect of ephrin-A5 is accompanied by an increasing sensitivity to the repulsive action of rostral Netrin-1. Future experiments will determine whether Netrin-1 is playing this function in large-scale mapping of ensembles of axons in other projection systems.

## Materials and Methods

### Animals.

Mice were used according to a protocol approved by the Institutional Animal Care and Use Committee at the University of North Carolina-Chapel Hill, and in accordance with National Institutes of Health guidelines. Time-pregnant females were maintained in a 12-h light/dark cycle and obtained by overnight breeding with males of the same strain. Noon following breeding is considered as E0.5. *Netrin-1* knockout mice (*Ntn1^Gt(pGT1.8TM)629Wcs^*, abbreviated *Ntn1^LacZ^*) were generated by crossing between heterozygous mice [21,55]. The initial line was on a mixed C57Bl6 and Sv129 background, and was backcrossed for more than ten generations on BALB/c background (Jackson Laboratories). Genotyping of *Netrin-1^LacZ^* mice was performed by the University of North Carolina genotyping core facility using quantitative PCR detecting the presence of zero, one, or two copies of the *lacZ* transgene. The genotype of embryos heterozygote or homozygote for the *Netrin-1* transgene was confirmed by anatomical defects described previously (absence of callosal and anterior commissure projections) [21]. Transgenic mice expressing EGFP under the control of CMV enhancer/chicken β-actin promoter were maintained by heterozygous crossing on a Balb/C background for more than ten generations [56].

### Biotinylated dextran amine (BDA) anterograde axon tracing in live mouse embryos.

Briefly, isolated hemispheres from E14.5 to E18.5 mouse embryos were microinjected (PicoSpritzer III; General Valve Corp.) using a medial approach with a 10% solution of lysine-fixable BDA (3,000 MW). Following incubation in oxygenated artificial cerebrospinal fluid (aCSF) for 5 h at 37 °C, the hemispheres were immersion fixed in 4% PFA. Injected hemispheres were sectioned coronally using a vibratome (LEICA VT1000S) at 100-μm thickness, permeabilized, and then incubated with Alexa546-conjugated streptavidin (1:1,000 in PBS + 0.1% Triton X-100 + 0.3% BSA) to reveal BDA. (See Figure S1.)

### RNA in situ hybridization.

Sense and antisense probes for mouse *Netrin-1*, *DCC*, *Unc5A*, *Unc5B* [27,57], and *Unc5C* [31,58] were generated as described previously. In situ hybridizations were performed as previously described using DIG-labeled probes [11].

### Immunofluorescent staining.

Whole-mount telencephalon/dorsal thalamic cocultures were maintained on organotypic slice culture inserts, fixed, and stained for immunofluorescence as previously described [10,59]. The following primary antibodies were used: polyclonal rabbit anti-β-galactosidase (1:1,000; Molecular Probes), monoclonal antineurofilament 165kD (clone 2H3; 1:2,000; Developmental Hybridoma Bank), polyclonal rat anti-L1 cell adhesion molecule (1:1,000; Chemicon) as well as polyclonal chicken and rabbit anti-GFP (1:2,000; Molecular Probes). The following secondary antibodies were used: Alexa-488, −546, and −647 conjugated goat anti-chicken, anti-rabbit, or anti-mouse IgG (1:2,000; Molecular Probes).

### Construction of Myc-tagged Unc5 cDNAs.

Full-length cDNA clones of mouse *Unc5A*, *Unc5B*, and *Unc5C* (Image ID: Unc5A, 6813463; Unc5B, 6417563; and Unc5C, 40085998) were purchased from Open Biosystems. The open reading frame of each clone was amplified by PCR using LA Taq polymerase (TAKARA BIO) and cloned with a Myc-tag at the carboxy-terminus into a modified pCIG2 vector, which drives expression of cloned cDNA from chicken ß-actin promoter and CMV enhancer. All constructs were confirmed by DNA sequencing.

### Western analysis.

COS7 cells were transfected with green fluorescent protein (GFP) and Unc5A-Myc, Unc5B-Myc, or Unc5C-Myc expression vectors using Lipofectamine 2000 (Invitrogen). Cells were lysed in RIPA buffer (50 mM Tris-Hcl [pH 7.5], 150 mM NaCl, 1% Triton X-100, 0.1% SDS) supplemented with protease inhibitors (Complete Protease Inhibitors Cocktail Tablets; Roche) 48 h after transfection. Protein samples (20 μg each) were run on 4%–12% gradient SDS-PAGE. gels (Invitrogen) and transferred to Hybond-P membranes (Amersham). Membranes were preincubated in 5% nonfat dry milk and 0.1% Tween-20 in Tris-buffered saline and incubated with the primary antibodies in the same solution. The primary antibodies used were mouse anti-Myc antibody (clone 9B11, 1:2,000; Cell Signaling Technology), goat anti-Unc5A antibody (anti-rat Unc5h1, 1:200; R&D Systems), and rabbit anti-GFP antibody (IgG fraction, 1:2,000; Molecular Probes). The fluorescent signals were generated using corresponding HRP-conjugated secondary antibodies and ECL-Plus Western Blotting Detection Reagents (Amersham), and were detected using a Typhoon 9400 image scanner (Amersham).

### Electroporation of plasmids into thalamic slices, axonal tracing, and quantification.

To visualize axons of dorsal thalamic neurons, we transfected myristoylated-Venus (mVenus) plasmid (pCX-myrVenus, kindly provided by Anna-Katerina Hadjantonakis, 1 μg/μl). For Unc5 overexpression, the mixture (0.5 μg/μl each) of Unc5A-Myc, Unc5B-Myc, and Unc5C-Myc (unpublished data) or Unc5C-Myc alone (1 μg/μl) was transfected together with mVenus plasmid (1 μg/μl). Control experiments included transfection of mVenus plasmid alone or the mixture of mVenus and Myc-tag empty plasmids. These two conditions gave similar results, and therefore, we combined them as control.

Electroporation into slices was performed essentially as described previously [37]. Briefly, coronal slices (250 μm) of E14.5 wild-type brains were prepared using a Leica VTel 1000S vibratome, and plasmid solution was pressure injected through a glass pipette into the rostral or caudal DTh (DTR or DTC; see Figure 10) using a Picrospritzer III (General Valve) microinjector. Electroporations were performed with gold-coated electrodes (GenePads 5 × 7 mm; BTX) using an ECM 830 electroporator (BTX) and the following parameters: five 5-ms–long pulses separated by 500-ms intervals at 100 V. After electroporation, the DTh was dissected and cocultured with E14.5 wild-type whole-mount telencephalon for 4 days, followed by fixation and immunostaining for GFP as described previously [10].

To identify GFP-positive axons, we used either of the two methods based on the axon density in each sample. When the number of axons in the sample was small, as shown in Figure 10D, we identified axons by single-axon tracing using the NeuronJ plug-in for ImageJ. Traced axons were superimposed on a reference framework with common origin, and the percentage of pixels in caudal, medial, or rostral 60° radial bin was quantified. When axon density in the sample was too high to identify individual axons, we quantified the percentage of EGFP-pixel distribution in the same way as in single-axon tracing. Thus, the two methods utilize essentially identical quantification analyses, and we combined the results obtained by using these two methods.

### Confocal microscopy.

Fluorescent immunostaining was observed using a LEICA TCS-SL laser scanning confocal microscope equipped with an Argon laser (488 nm), green helium-neon laser (546 nm), and red helium-neon laser line (633 nm) mounted on an inverted DM-IRE2 microscope equipped with a Marzhauzer X-Y motorized stage allowing large-scale tiling of whole-mount telencephalic cocultures obtained by scanning multiple fields using a long working distance 10× objective followed by an automatic tiling function available from the LEICA confocal software.

## Supporting Information

Figure S1 Biotinylated Dextran Amine (BDA) Axon Tracing and Reconstruction of the Topography of Thalamocortical Projections in Embryonic Mouse Brain (A) Experimental paradigm underlying BDA anterograde tracing in live mouse embryonic DTh.
(B–D′) This method allows the labeling of small numbers of thalamic neurons as visualized on these three confocal micrographs taken of adjacent 100-μm–thick vibratome sections of the anterior portion of the thalamus of an E14.5 hemisphere (injection site delineated by red shaded area in [B′–D′]). This method allows the full anterograde labeling of thalamic axons (red arrow points to growth cone in the internal capsule shown in [E]) and is compatible with immunofluorescent staining (neurofilament 165kD staining in green in [B–D]). (B–D) are counterstained with the axonal marker neurofilament 165kD (green) and nucleic acid staining DRAQ5 (blue).
(F–S) Image analysis involved in the reconstruction of the topography of TC projections at the level of the CSB. Steps 1 and 2: individual BDA microinjections in the DTh of single E18.5 mouse hemisphere (F) and its resulting axon projection (E) are traced onto corresponding DRAQ5-counterstained coronal sections ([G and H]; see also Figure S2 for the entire “model” coronal section series). Steps 3 and 4: extraction of the *X-Y* coordinates of the injection site (K) and the axon tracts (J) on a mask of model sections. Step 4: averaging of the traces of multiple axon projections (L) resulting from multiple injections sites ([M]; see Figure 2 for different categorization of injection sites). This is performed in coronal sections where anatomical regions such as the DTh (green outline in [L]) or the CSB (yellow outline in [K and L]) correspond to individual masks (see also Figure S2). Steps 5 and 6: categorized and averaged axon traces crossing the CSB at each section are masked (M and N), linearized, and aligned (O) in a frame organized along the rostrocaudal (R-C) axis (vertical) and the dorsoventral (D-V) axis (horizontal). Finally, in Steps 7 and 8, the individual CSB frames are assembled in a common referenced space (R) and then normalized along the R-C and the D-V axes (S).
(T) Schematic representation of the position of the CSB reconstructions shown in (S) on a horizontal section of the mouse brain.
(2.61 MB TIF)

Figure S2 Regional Segmentation of E18.5 Mouse Brain Used for Reconstruction of Biotinylated Dextran Amine Axon Tracing (A) Series of adjacent 100-μm–thick coronal sections of an E18.5 mouse brain used as a model for BDA axon tracing and injection site reconstruction. Sections numbered from 1 to 34 (rostral to caudal) were counterstained with DRAQ5, revealing the cytoarchitecture of distinct regions outlined in (B).
(B) Segmentation of distinct regions used for reconstructions as shown in Figure 1. Red indicates cortex; yellow, corticostriatal boundary; green, DTh; blue, ganglionic eminence (VTel); and pink, hippocampus.
(2.41 MB TIF)

Figure S3 The Topography of TC Axon Projections Is Established at E15 in the Mouse Ventral Telencephalon BDA microinjection was performed in the DTh of multiple E15.5 mouse embryos, and axon tracing analysis was performed as in Figure 1.
(A and A′) Axon density maps of TC projections at the CSB for axons originating along the rostrocaudal axis of the DTh (A′). Color code corresponds to injections performed in three-thirds of the DTh along the rostrocaudal axis of the DTh shown individually in (A1–A3).
(B and B′) Same analysis as in (A and A′) but along the mediolateral axis of the DTh at E15.5.
(847 KB TIF)

Figure S4 Absence of Obvious Thalamocortical Axon Pathfinding Defect in the Internal Capsule of the *Netrin-1* Knockout Mouse at E18.5 Confocal reconstruction of immunofluorescent staining for the axonal marker L1 on 100-μm–thick horizontal sections taken at 400-μm intervals of a wild-type (A–D) and a *Netrin-1* knockout (E–H) E17.5 mouse embryo. L1 stains both TC axons as well as other axon tracts such as the corpus callosum (CC), but not other corticofugal axons ([25]; A. Powell and F. Polleux, unpublished data). Red arrowheads indicate the internal capsule, and green arrows indicate the thalamic peduncle. CC and anterior commissure projection defects can be observed in the *Netrin-1* knockout embryos as described previously [27]. However, no gross TC axon pathfinding defect can be detected at this level. VTh, ventral thalamus.
Scale bar represents 1 mm.
(1.39 MB TIF)

Figure S5 Disrupted Topography of Thalamocortical Projections Achieved at the Level of the Ventral Telencephalon of the *Netrin-1* Knockout This figure is essentially the same as Figure 1 but for *Netrin-1* knockout E18.5 embryos.
(A) Averaged axon density maps quantified from multiple BDA injections (*n* numbers in A1–A3) clustered in three, arbitrarily defined thirds along the rostrocaudal axis of the E18.5 *Netrin-1* ^−/−^ mouse DTh as done in Figure 1 for wild-type control embryos (red indicates rostral; green, medial; and blue, caudal; as shown in [A′]). (A1–A3) Individual average axon density maps for thalamic injections clustered in the rostral- (A1), medial- (A2), or caudal-most (A3) third of *Netrin-1* ^−/−^ DTh.
(B) Averaged axon density maps quantified from multiple BDA injections clustered along the mediolateral axis of the DTh (red indicates medial; green, central; and blue, lateral; as shown in [B′]).
(C) Averaged axon density maps shown in (A1) (rostral-most third of DTh split in lateral and medial halves), (A2) (medial third along rostrocaudal extent), and (A3) (caudal third along rostrocaudal extent) were further subdivided into halves (C1) or thirds (C2 and C3) along the mediolateral axis. This analysis demonstrates the significant lack of topographic segregation of thalamic axon projections characterizing the *Netrin-1* ^−/−^ E18.5 embryos.
(D and D′) Averaged position of BDA injection sites in the DTh leading to axons crossing CSB at its most rostral (red), medial (green), or caudal-most (blue) third in *Netrin-1* ^−/−^ knockout embryos at E18.5. This 2-D map represents a dorsal view of the DTh, compressed along its dorsoventral axis.
(D1–D3) Individual averaged density maps of thalamic injection sites leading to axons crossing the CSB at its rostral- (D1), medial- (D2), or caudal-most (D3) third. The arrowheads point to the domain of the DTh projecting abnormally compared to wild-type embryos (see Figure 1D1-1D3 for comparison).
(E) Schematic representation of the anatomical location of our 2-D averaged axon density maps as shown in Figure 1.
(3.34 MB TIF)

Figure S6 Statistical Analysis of the Differences in the Averaged Axon Density Maps of Thalamocortical Projections between Wild-Type and *Netrin-1* Knockout Embryos Each map shown in Figure 4I–4S comparing the distribution of TC projections at the CSB between wild-type and *Netrin-1* ^−/−^ embryos have been divided into 12 bins along the rostrocaudal and dorsoventral axis of the CSB (A–H) or 25 bins in the DTh (I–L). Within each bin, two-way ANOVA test was used to determine the significance of the axon density maps between wild-type (WT) and *Netrin-1* knockout embryos. Significance was arbitrarily set at *p* < 0.001, with green representing bins in which the averaged density observed in WT is superior to *Netrin-1* ^−/−^, and vice versa for red bins. Any comparison with *p* > 0.001 was considered nonsignificant and shown in white.
(1.61 MB TIF)

Figure S7 Differences of TC Projections along the Dorsoventral Axis of the Ventral Telencephalon between Wild-Type and *Netrin-1* Knockout Embryos (A–C) Superimposition of the averaged injection site position in the DTh of wild-type (green) and *Netrin-1* ^−/−^ (red) embryos for thalamic axons crossing the CSB along its dorsal (B) or ventral (C) halves as shown in (A).
(D–F) Statistical analysis using a two-way ANOVA test to determine the significance of the density maps between wild-type (WT) and *Netrin-1* knockout embryos shown in (B and C). Significance was arbitrarily set at *p* < 0.001 with green representing bins in which the averaged density observed in WT is superior to *Netrin-1* ^−/−^ and vice versa for red bins. Any comparison with p > 0.001 was considered nnonsignificant and shown in white.
(1.17 MB TIF)

Figure S8 Analysis of the Length of the Longest Axon Projecting in Telencephalic Whole Mount Reveals That Netrin-1 Is Not Required for DTh Axon Outgrowth The length of the longest thalamic axon was measured in whole-mount cocultures shown in Figure 5 between wild-type EGFP^+^ DTR or DTC explants and either wild-type (WT-VTel) or *Netrin-1* ^−/−^ (*Netrin-1* ^−/−^ VTel) telencephalon. This analysis reveals no significant differences (p > 0.05 according to nonparametric Mann-Whitney test) between the length of the longest DTR or DTC axon growing in wild-type or Netrin-1–deficient VTel, suggesting that Netrin-1 is not required in vivo for DTh axon extension.
(2.89 MB TIF)

Figure S9 Differential Effect of Netrin-1 on the Guidance of Rostromedial and Caudolateral Thalamic Axons In Vitro Collagen cocultures of DTR axons (A and B) and DTC axons (D and E) with either control HEK 293 (A and D) or 293 cells stably expressing Netrin-1 (B and E). Axons are visualized using anti-Neurofilament 165kD immunofluorescence (green). Blue is DRAQ5 nuclear staining. Quantification of the orientation of axon outgrowth categorized as growing towards, symmetrically, or away from the 293 cell aggregates as described in [22]. DTR axons show a strong attraction towards Netrin-1–expressing cells, but not control HEK cells, whereas DTC axons show a modest (but significantly different from control) repulsion from Netrin-1–expressing cell aggregates. Chi-square analysis: DTR–Control 293 cells versus Netrin-1 cells 293, *p* < 0.001; DTC–Control 293 cells versus Netrin-1 293 cells, *p* < 0.01; DTR versus DTC to Netrin-1 293 cells, *p* < 0.001.
(365 KB TIF)

Figure S10 Complementary Expression Patterns of *DCC* and *Unc5A* and *B* Receptors in the Dorsal Thalamus, Ventral Thalamus, and Epithalamus of E14.5 Mouse Embryos (A–L) mRNA in situ hybridization for *DCC* ([A–C], pseudo-colored green in [J–L]), Unc5B ([D–F], pseudo-colored red in [J–]L), and *Unc5A* ([G–I], pseudo-colored blue in [J–L]), performed on coronal sections of E14.5 mouse embryos reveals that in addition to their expression in the DTh, DCC and Unc5B are also expressed at high levels in the ventral thalamus (B, E, and K) and the epithalamus (B, C, K, and L), respectively. The arrowheads in (A–C) point to the rostromedial domain of high expression for DCC, whereas the arrows in (C) represent the caudolateral low-level expression of DCC. Each individual panel represents in situ hybridization performed using DIG-labeled probes on adjacent 20-μm–thick coronal sections. Original bright-field images captured with a charge-coupled device (CCD) camera were inverted in order to be merged in Adobe Photoshop (version 9.0) using a pseudo-coloring RGB function.
(3.4 MB TIF)

Figure S11 Microdissection of Thalamic Explants and Their Relationship to Netrin-1 Receptor Expression Patterns Dorsal thalamic explants are isolated on adjacent 250-μm–thick vibratome sections performed on E14.5 EGFP^+^ mouse embryos (top panel, ordered from rostral to more-caudal levels going from left to right). Expression patterns of *DCC* and *Unc5C* receptors, shown in Figures 7 and S10, suggest that section 1 (systematically used for DTR explants [10]) expresses high levels of DCC receptor, whereas section 4 (systematically used for DTC explants [10]) expresses low levels of DCC and high levels of Unc5A–C.
(440 KB TIF)

Figure S12 Biochemical Analysis of the Reactivity of Function-Blocking Anti-Unc5A COS7 cells were transfected with myc-tagged Unc5A, Unc5B, or Unc5C expressed under chicken β-actin promoter (pCIG2) followed by IRES-mVenus. Two days after transfection, cells were lysed and lysates subjected to SDS-PAGE and probed with mouse anti-Myc antibody (clone 9B11, 1:2,000; Cell Signaling Technology), goat anti-Unc5A antibody (anti-rat Unc5H1, 1:200; R&D Systems), and rabbit anti-GFP antibody (IgG fraction, 1:2,000; Molecular Probes). The fluorescent signals were detected using Typhoon 9400 image scanner (Amersham) in the linear range. Ratios indicate the relative fluorescence obtained with anti-Unc5A (H1) and anti-myc (control for amount of recombinant Unc5A-B-C protein present in lysate). This analysis demonstrates that this commercially available anti-Unc5A cross-reacts with Unc5C (but not Unc5B) and has approximately three times more affinity for Unc5A than for Unc5C.
(202 KB TIF)

## Abbreviations

ANOVA: analysis of variance
BDA: biotinylated dextran amine
CSB: corticostriatal boundary
DTC: caudal dorsal thalamus
DTh: dorsal thalamus
DTR: rostral dorsal thalamus
E: embryonic day
TC: thalamocortical
VTel: ventral telencephalon
