# Supplementary figures and images for: Topography of Thalamic Projections Requires Attractive and Repulsive Functions of Netrin-1 in the Ventral Telencephalon
Source: PLoS Biol. 2008 May 13;6(5):e116. doi: 10.1371/journal.pbio.0060116 (PMC2584572; doi:10.1371/journal.pbio.0060116)

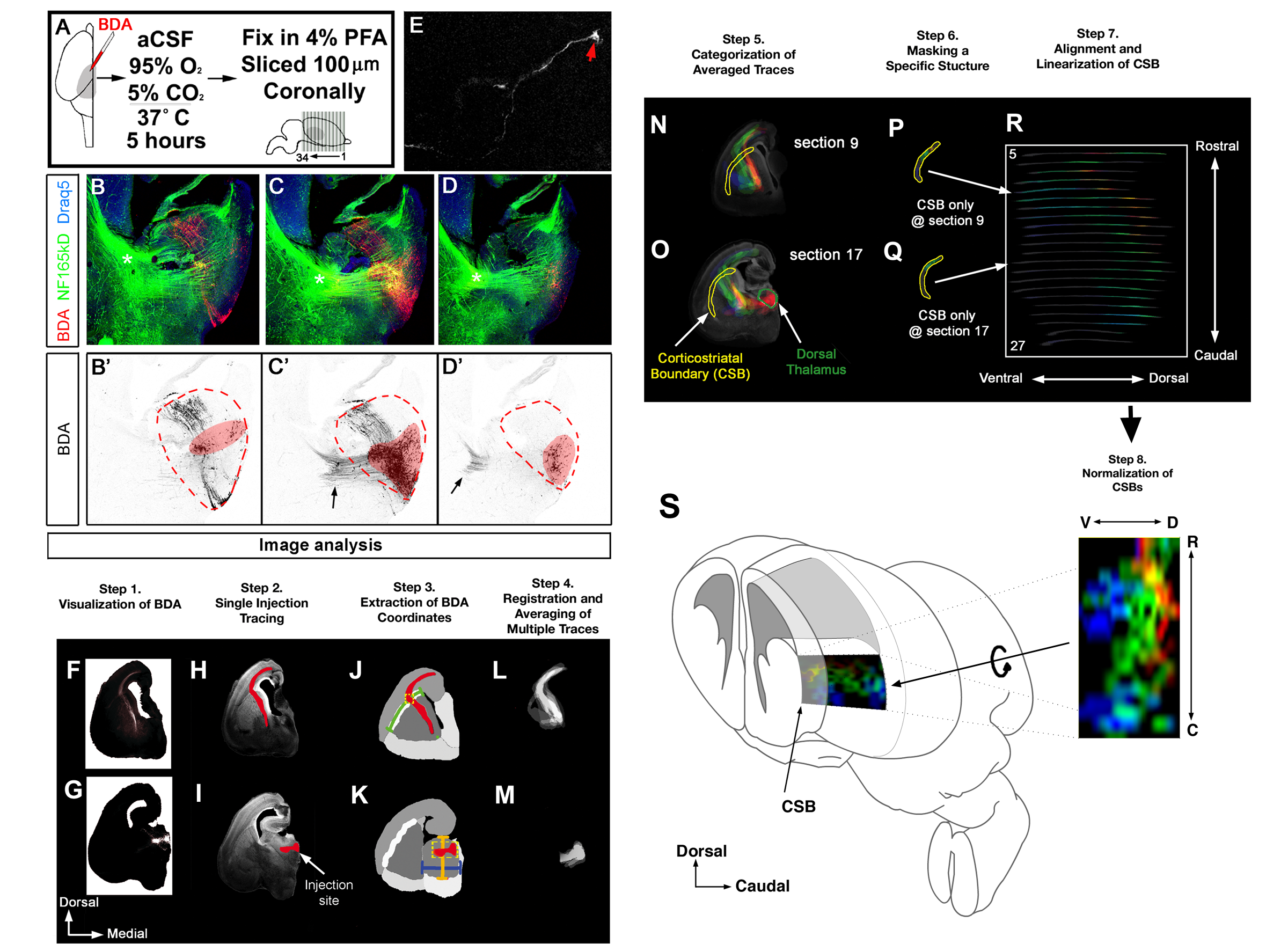

Supplement: Figure S1 — (A) Experimental paradigm underlying BDA anterograde tracing in live mouse embryonic DTh. (B–D′) This method allows the labeling of small numbers of thalamic neurons as visualized on these three confocal micrographs taken of adjacent 100-μm–thick vibratome sections of the anterior portion of the thalamus of an E14.5 hemisphere (injection site delineated by red shaded area in [B′–D′]). This method allows the full anterograde labeling of thalamic axons (red arrow points to growth cone in the internal capsule shown in [E]) and is compatible with immunofluorescent staining (neurofilament 165kD staining in green in [B–D]). (B–D) are counterstained with the axonal marker neurofilament 165kD (green) and nucleic acid staining DRAQ5 (blue). (F–S) Image analysis involved in the reconstruction of the topography of TC projections at the level of the CSB. Steps 1 and 2: individual BDA microinjections in the DTh of single E18.5 mouse hemisphere (F) and its resulting axon projection (E) are traced onto corresponding DRAQ5-counterstained coronal sections ([G and H]; see also Figure S2 for the entire “model” coronal section series). Steps 3 and 4: extraction of the X-Y coordinates of the injection site (K) and the axon tracts (J) on a mask of model sections. Step 4: averaging of the traces of multiple axon projections (L) resulting from multiple injections sites ([M]; see Figure 2 for different categorization of injection sites). This is performed in coronal sections where anatomical regions such as the DTh (green outline in [L]) or the CSB (yellow outline in [K and L]) correspond to individual masks (see also Figure S2). Steps 5 and 6: categorized and averaged axon traces crossing the CSB at each section are masked (M and N), linearized, and aligned (O) in a frame organized along the rostrocaudal (R-C) axis (vertical) and the dorsoventral (D-V) axis (horizontal). Finally, in Steps 7 and 8, the individual CSB frames are assembled in a common referenced space (R) and then normalized [file pbio.0060116.sg001.tif]

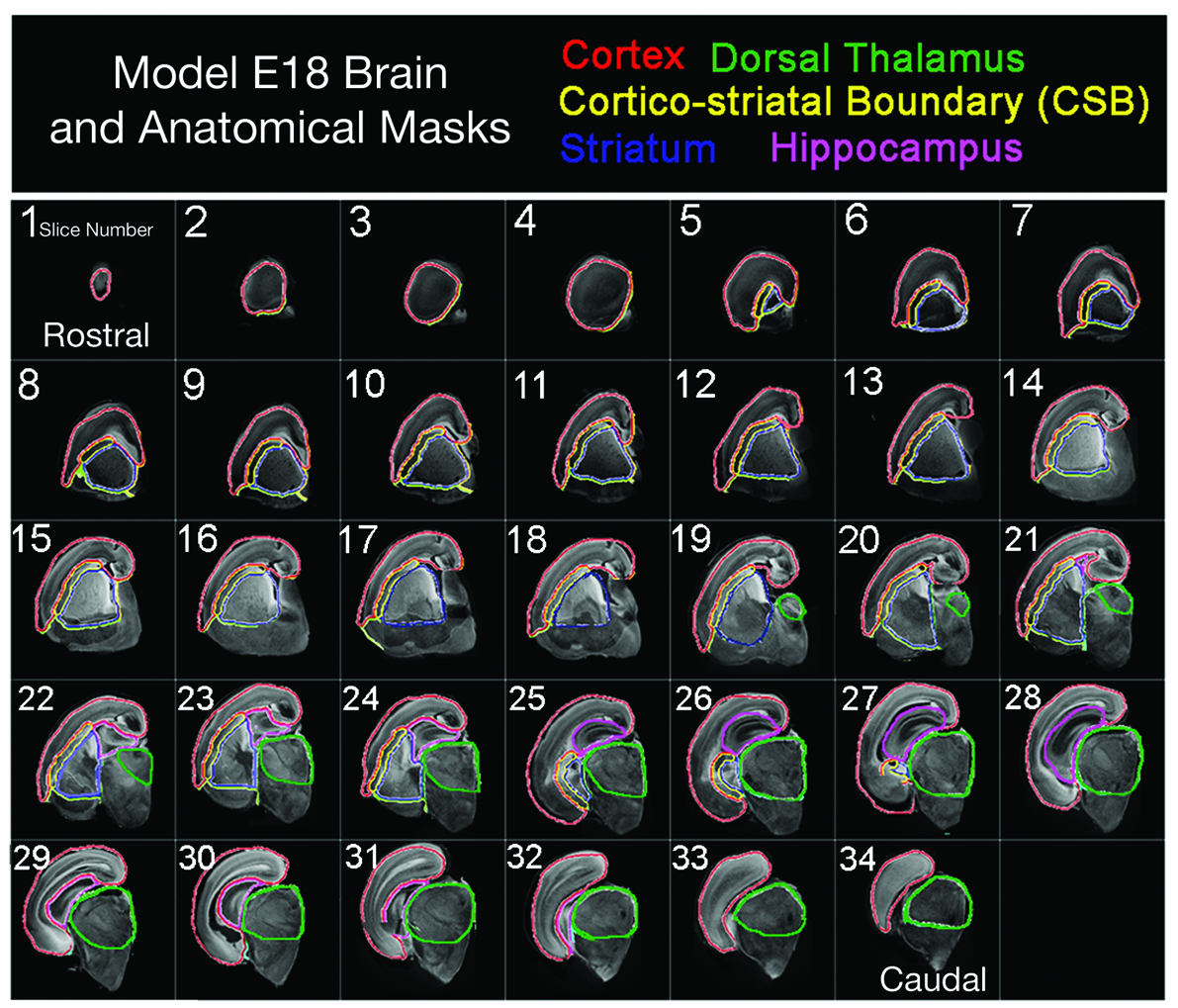

Supplement: Figure S2 — (A) Series of adjacent 100-μm–thick coronal sections of an E18.5 mouse brain used as a model for BDA axon tracing and injection site reconstruction. Sections numbered from 1 to 34 (rostral to caudal) were counterstained with DRAQ5, revealing the cytoarchitecture of distinct regions outlined in (B). (B) Segmentation of distinct regions used for reconstructions as shown in Figure 1. Red indicates cortex; yellow, corticostriatal boundary; green, DTh; blue, ganglionic eminence (VTel); and pink, hippocampus. (2.41 MB TIF) [file pbio.0060116.sg002.tif]

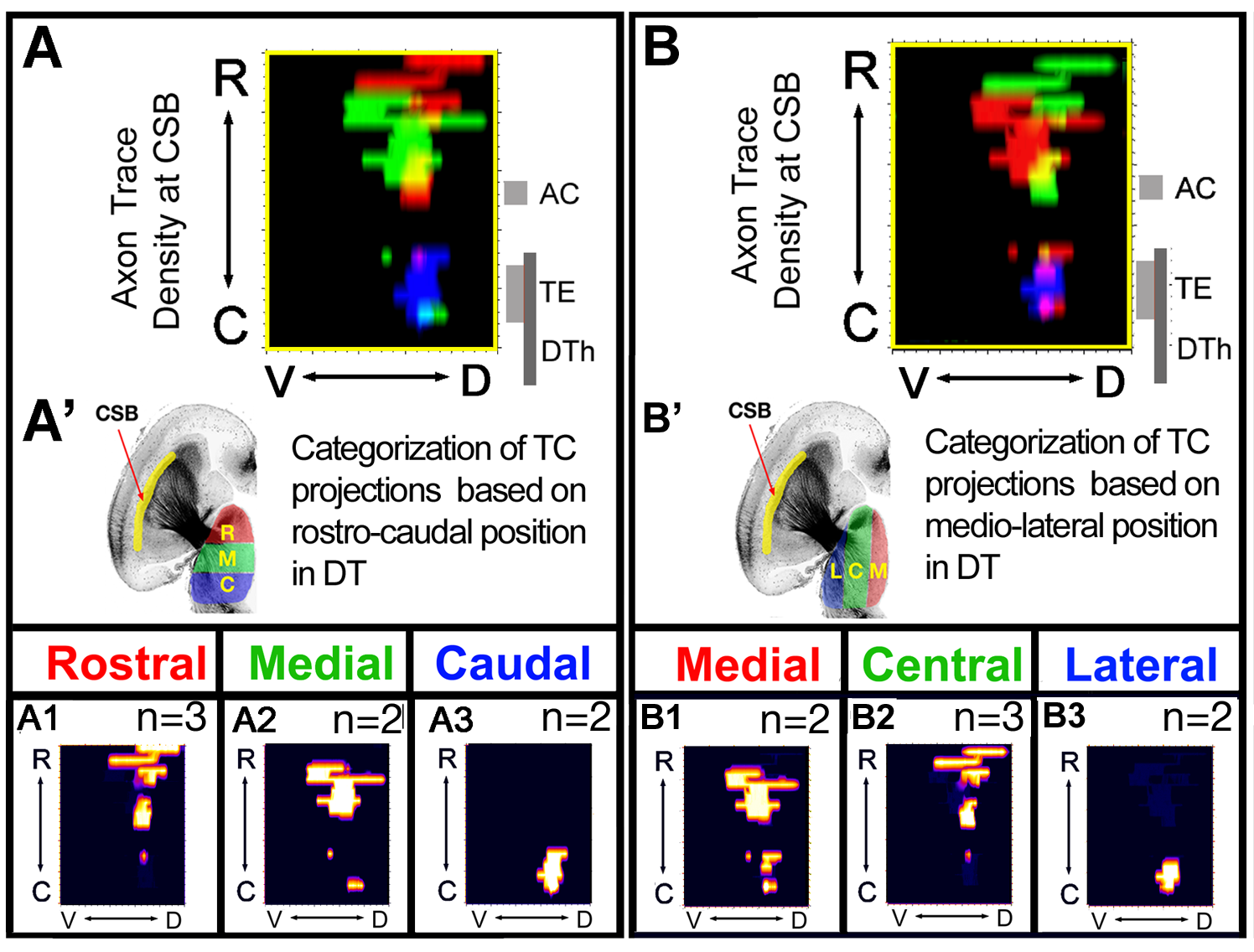

Supplement: Figure S3 — BDA microinjection was performed in the DTh of multiple E15.5 mouse embryos, and axon tracing analysis was performed as in Figure 1. (A and A′) Axon density maps of TC projections at the CSB for axons originating along the rostrocaudal axis of the DTh (A′). Color code corresponds to injections performed in three-thirds of the DTh along the rostrocaudal axis of the DTh shown individually in (A1–A3). (B and B′) Same analysis as in (A and A′) but along the mediolateral axis of the DTh at E15.5. (847 KB TIF) [file pbio.0060116.sg003.tif]

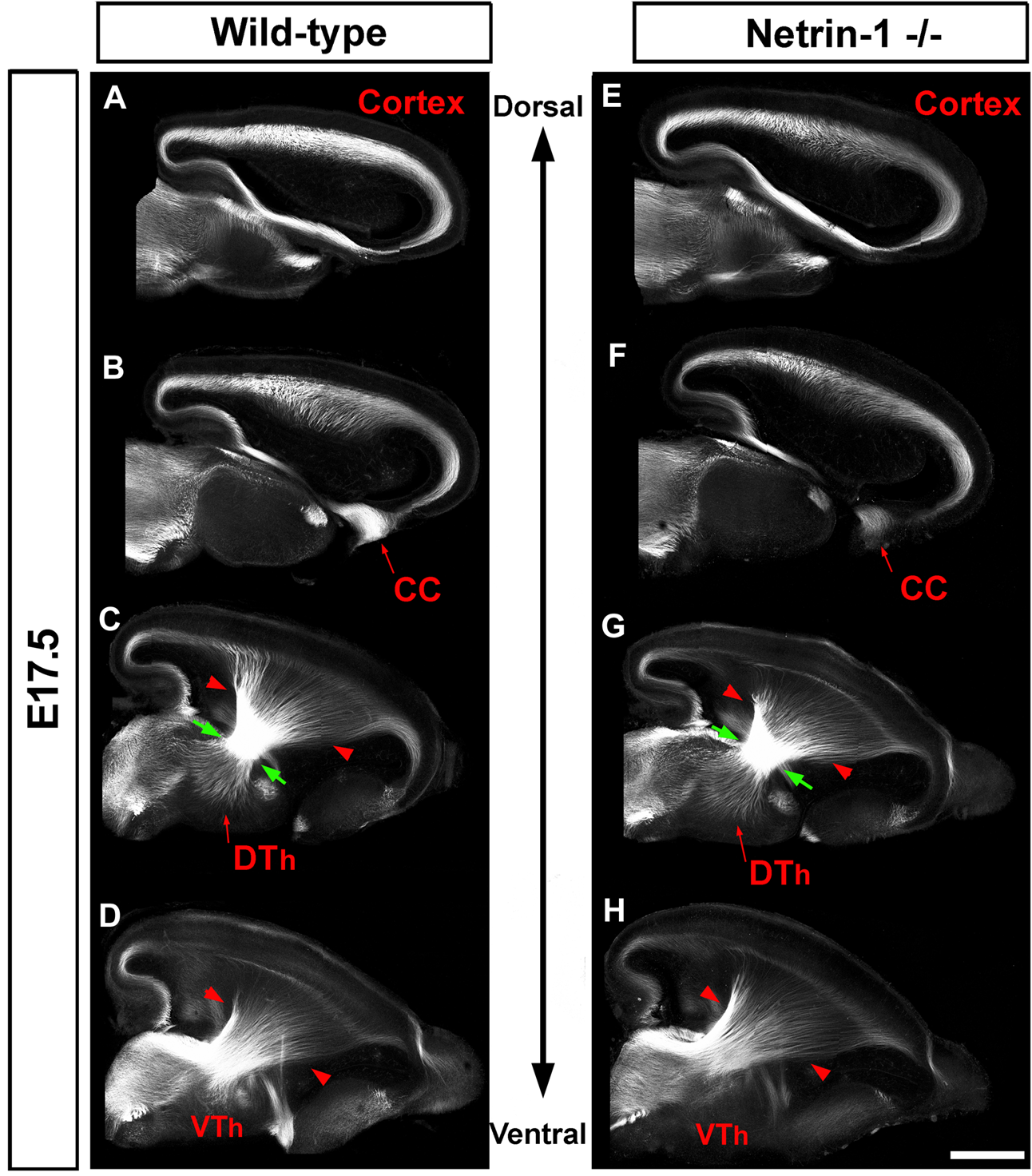

Supplement: Figure S4 — Confocal reconstruction of immunofluorescent staining for the axonal marker L1 on 100-μm–thick horizontal sections taken at 400-μm intervals of a wild-type (A–D) and a Netrin-1 knockout (E–H) E17.5 mouse embryo. L1 stains both TC axons as well as other axon tracts such as the corpus callosum (CC), but not other corticofugal axons ([25]; A. Powell and F. Polleux, unpublished data). Red arrowheads indicate the internal capsule, and green arrows indicate the thalamic peduncle. CC and anterior commissure projection defects can be observed in the Netrin-1 knockout embryos as described previously [27]. However, no gross TC axon pathfinding defect can be detected at this level. VTh, ventral thalamus. Scale bar represents 1 mm. (1.39 MB TIF) [file pbio.0060116.sg004.tif]

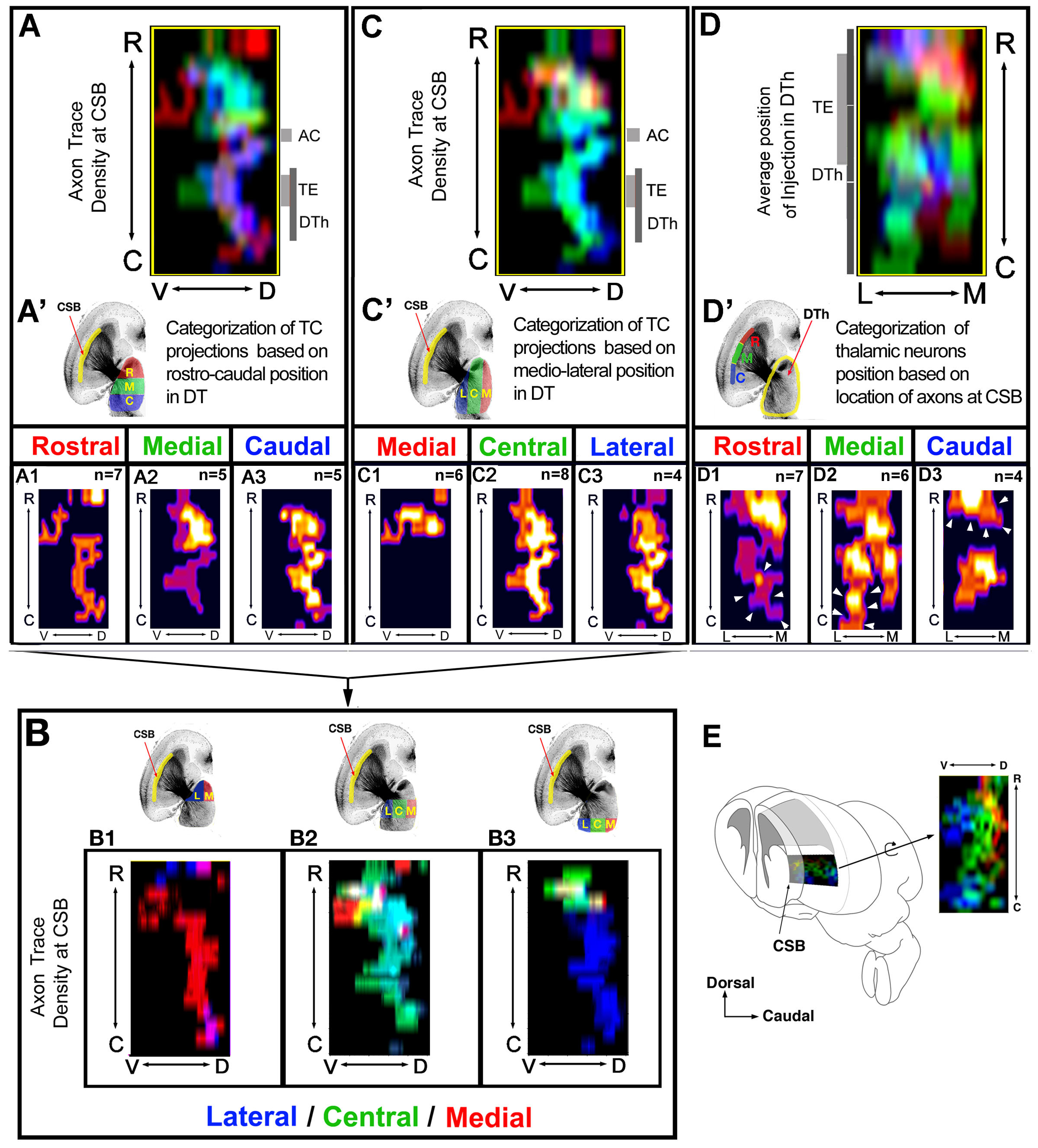

Supplement: Figure S5 — This figure is essentially the same as Figure 1 but for Netrin-1 knockout E18.5 embryos. (A) Averaged axon density maps quantified from multiple BDA injections (n numbers in A1–A3) clustered in three, arbitrarily defined thirds along the rostrocaudal axis of the E18.5 Netrin-1 −/− mouse DTh as done in Figure 1 for wild-type control embryos (red indicates rostral; green, medial; and blue, caudal; as shown in [A′]). (A1–A3) Individual average axon density maps for thalamic injections clustered in the rostral- (A1), medial- (A2), or caudal-most (A3) third of Netrin-1 −/− DTh. (B) Averaged axon density maps quantified from multiple BDA injections clustered along the mediolateral axis of the DTh (red indicates medial; green, central; and blue, lateral; as shown in [B′]). (C) Averaged axon density maps shown in (A1) (rostral-most third of DTh split in lateral and medial halves), (A2) (medial third along rostrocaudal extent), and (A3) (caudal third along rostrocaudal extent) were further subdivided into halves (C1) or thirds (C2 and C3) along the mediolateral axis. This analysis demonstrates the significant lack of topographic segregation of thalamic axon projections characterizing the Netrin-1 −/− E18.5 embryos. (D and D′) Averaged position of BDA injection sites in the DTh leading to axons crossing CSB at its most rostral (red), medial (green), or caudal-most (blue) third in Netrin-1 −/− knockout embryos at E18.5. This 2-D map represents a dorsal view of the DTh, compressed along its dorsoventral axis. (D1–D3) Individual averaged density maps of thalamic injection sites leading to axons crossing the CSB at its rostral- (D1), medial- (D2), or caudal-most (D3) third. The arrowheads point to the domain of the DTh projecting abnormally compared to wild-type embryos (see Figure 1D1-1D3 for comparison). (E) Schematic representation of the anatomical location of our 2-D averaged axon density maps as shown in Figure 1. (3.34 MB TIF) [file pbio.0060116.sg005.tif]

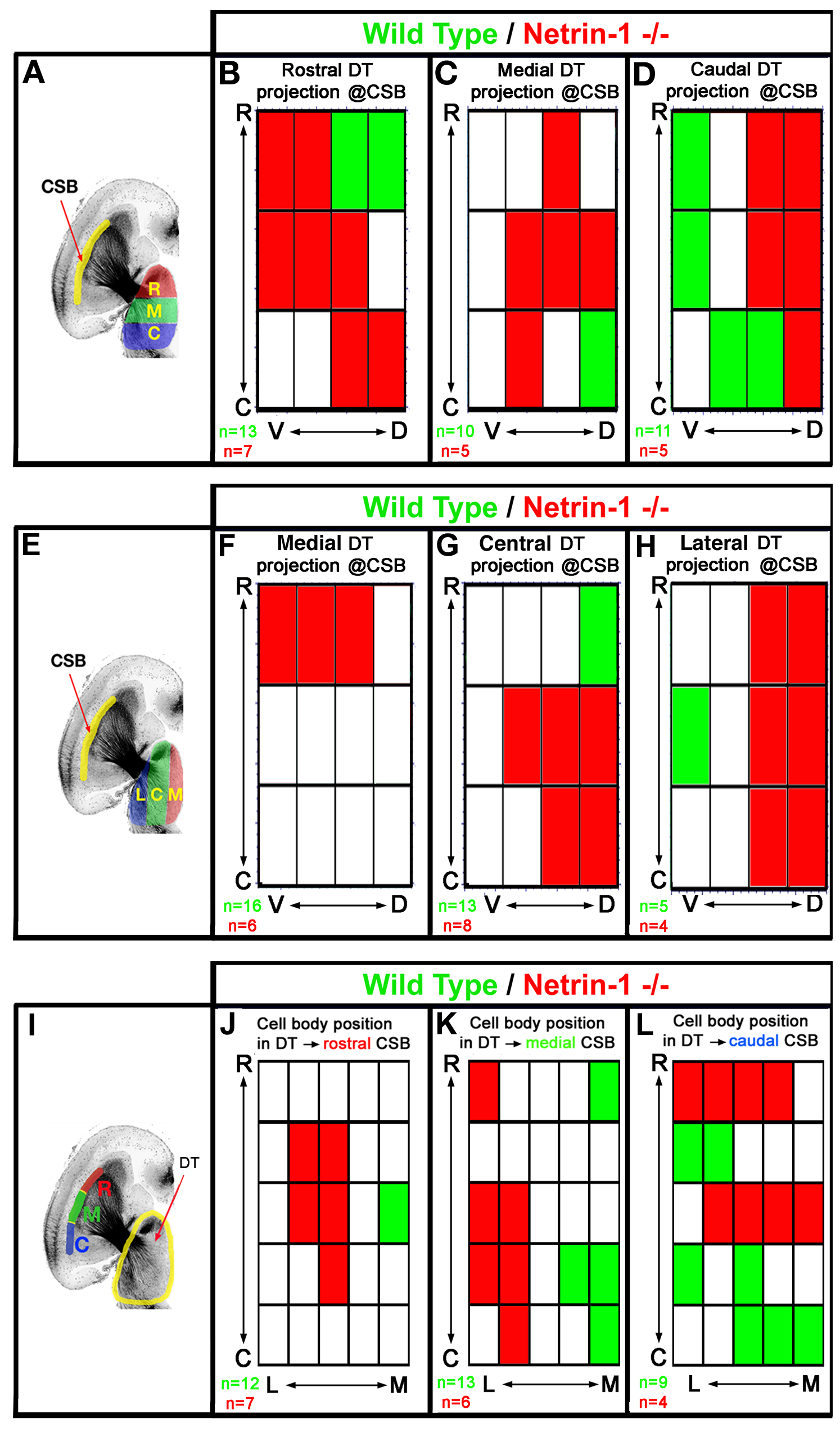

Supplement: Figure S6 — Each map shown in Figure 4I–4S comparing the distribution of TC projections at the CSB between wild-type and Netrin-1 −/− embryos have been divided into 12 bins along the rostrocaudal and dorsoventral axis of the CSB (A–H) or 25 bins in the DTh (I–L). Within each bin, two-way ANOVA test was used to determine the significance of the axon density maps between wild-type (WT) and Netrin-1 knockout embryos. Significance was arbitrarily set at p < 0.001, with green representing bins in which the averaged density observed in WT is superior to Netrin-1 −/−, and vice versa for red bins. Any comparison with p > 0.001 was considered nonsignificant and shown in white. (1.61 MB TIF) [file pbio.0060116.sg006.tif]

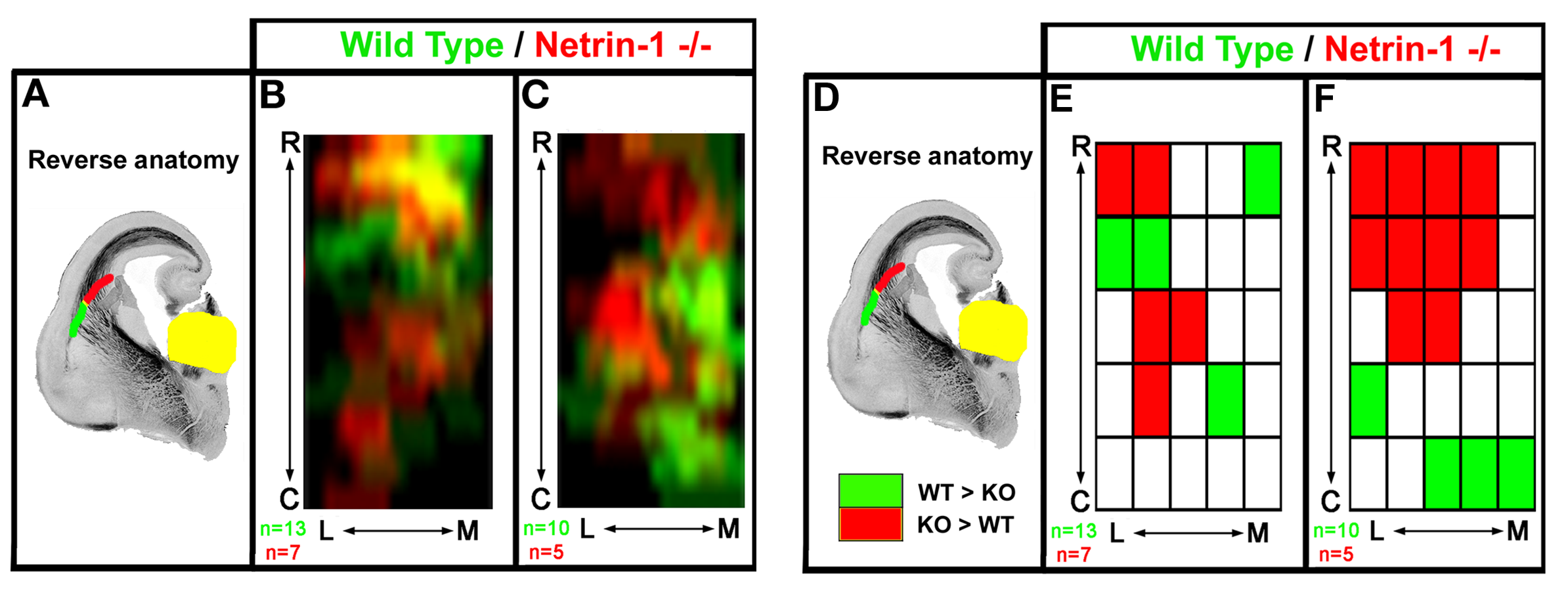

Supplement: Figure S7 — (A–C) Superimposition of the averaged injection site position in the DTh of wild-type (green) and Netrin-1 −/− (red) embryos for thalamic axons crossing the CSB along its dorsal (B) or ventral (C) halves as shown in (A). (D–F) Statistical analysis using a two-way ANOVA test to determine the significance of the density maps between wild-type (WT) and Netrin-1 knockout embryos shown in (B and C). Significance was arbitrarily set at p < 0.001 with green representing bins in which the averaged density observed in WT is superior to Netrin-1 −/− and vice versa for red bins. Any comparison with p > 0.001 was considered nnonsignificant and shown in white. (1.17 MB TIF) [file pbio.0060116.sg007.tif]

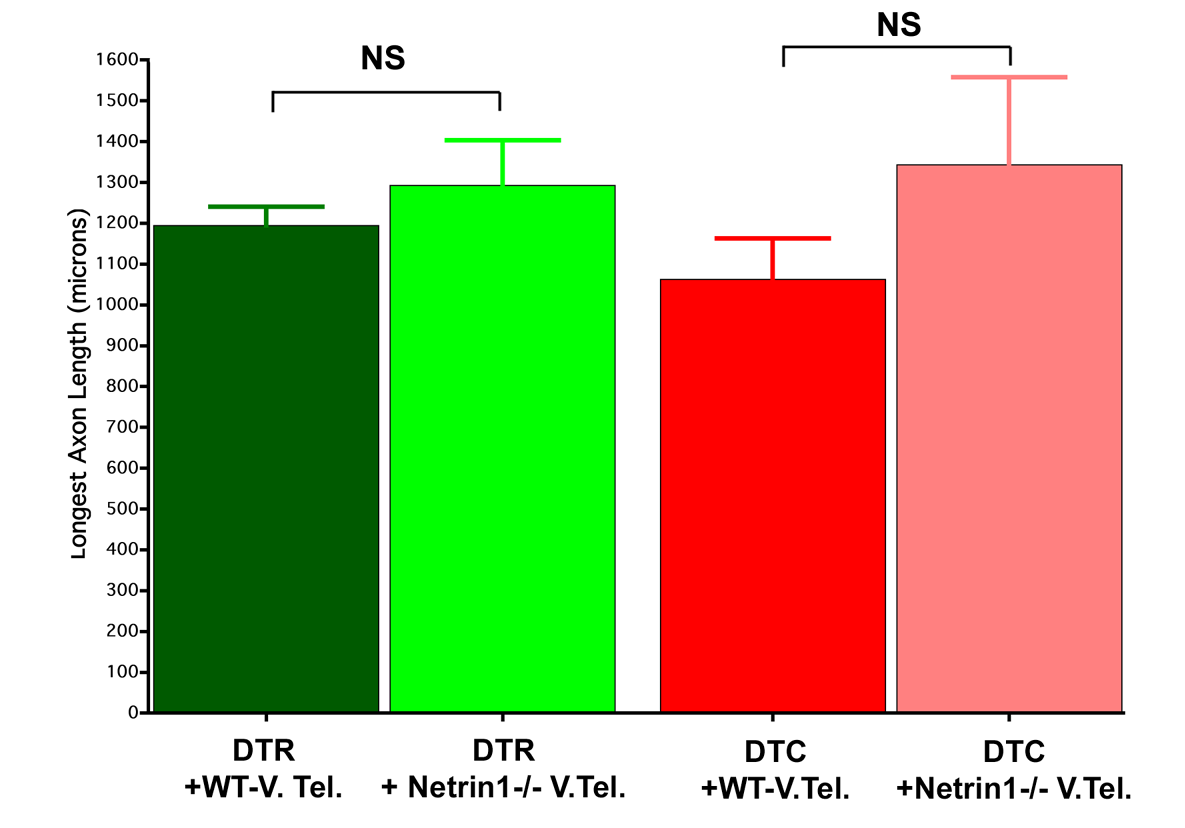

Supplement: Figure S8 — The length of the longest thalamic axon was measured in whole-mount cocultures shown in Figure 5 between wild-type EGFP+ DTR or DTC explants and either wild-type (WT-VTel) or Netrin-1 −/− (Netrin-1 −/− VTel) telencephalon. This analysis reveals no significant differences (p > 0.05 according to nonparametric Mann-Whitney test) between the length of the longest DTR or DTC axon growing in wild-type or Netrin-1–deficient VTel, suggesting that Netrin-1 is not required in vivo for DTh axon extension. (2.89 MB TIF) [file pbio.0060116.sg008.tif]

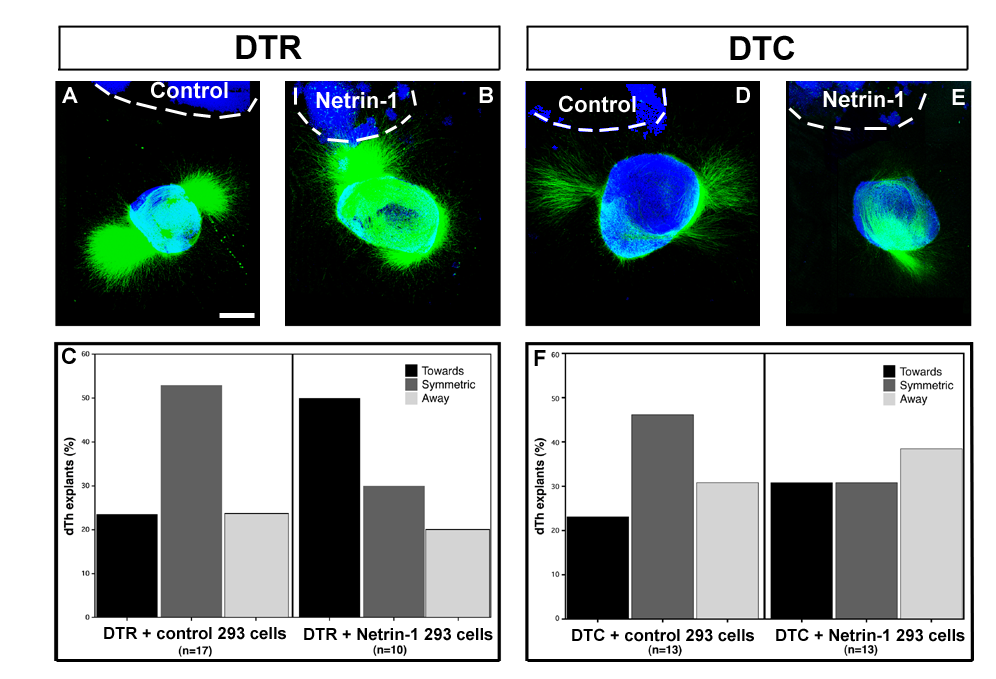

Supplement: Figure S9 — Collagen cocultures of DTR axons (A and B) and DTC axons (D and E) with either control HEK 293 (A and D) or 293 cells stably expressing Netrin-1 (B and E). Axons are visualized using anti-Neurofilament 165kD immunofluorescence (green). Blue is DRAQ5 nuclear staining. Quantification of the orientation of axon outgrowth categorized as growing towards, symmetrically, or away from the 293 cell aggregates as described in [22]. DTR axons show a strong attraction towards Netrin-1–expressing cells, but not control HEK cells, whereas DTC axons show a modest (but significantly different from control) repulsion from Netrin-1–expressing cell aggregates. Chi-square analysis: DTR–Control 293 cells versus Netrin-1 cells 293, p < 0.001; DTC–Control 293 cells versus Netrin-1 293 cells, p < 0.01; DTR versus DTC to Netrin-1 293 cells, p < 0.001. (365 KB TIF) [file pbio.0060116.sg009.tif]

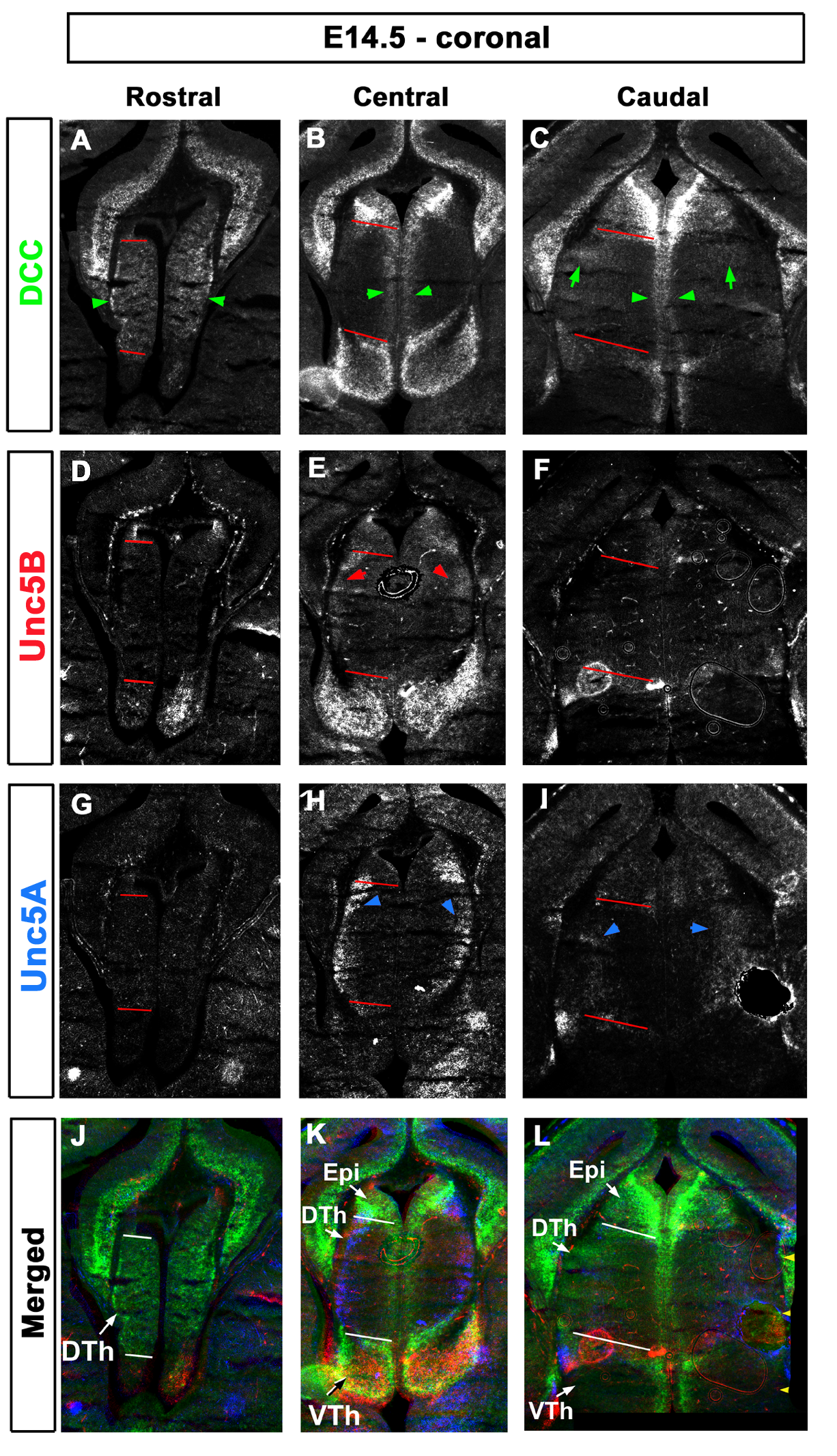

Supplement: Figure S10 — (A–L) mRNA in situ hybridization for DCC ([A–C], pseudo-colored green in [J–L]), Unc5B ([D–F], pseudo-colored red in [J–]L), and Unc5A ([G–I], pseudo-colored blue in [J–L]), performed on coronal sections of E14.5 mouse embryos reveals that in addition to their expression in the DTh, DCC and Unc5B are also expressed at high levels in the ventral thalamus (B, E, and K) and the epithalamus (B, C, K, and L), respectively. The arrowheads in (A–C) point to the rostromedial domain of high expression for DCC, whereas the arrows in (C) represent the caudolateral low-level expression of DCC. Each individual panel represents in situ hybridization performed using DIG-labeled probes on adjacent 20-μm–thick coronal sections. Original bright-field images captured with a charge-coupled device (CCD) camera were inverted in order to be merged in Adobe Photoshop (version 9.0) using a pseudo-coloring RGB function. (3.4 MB TIF) [file pbio.0060116.sg010.tif]

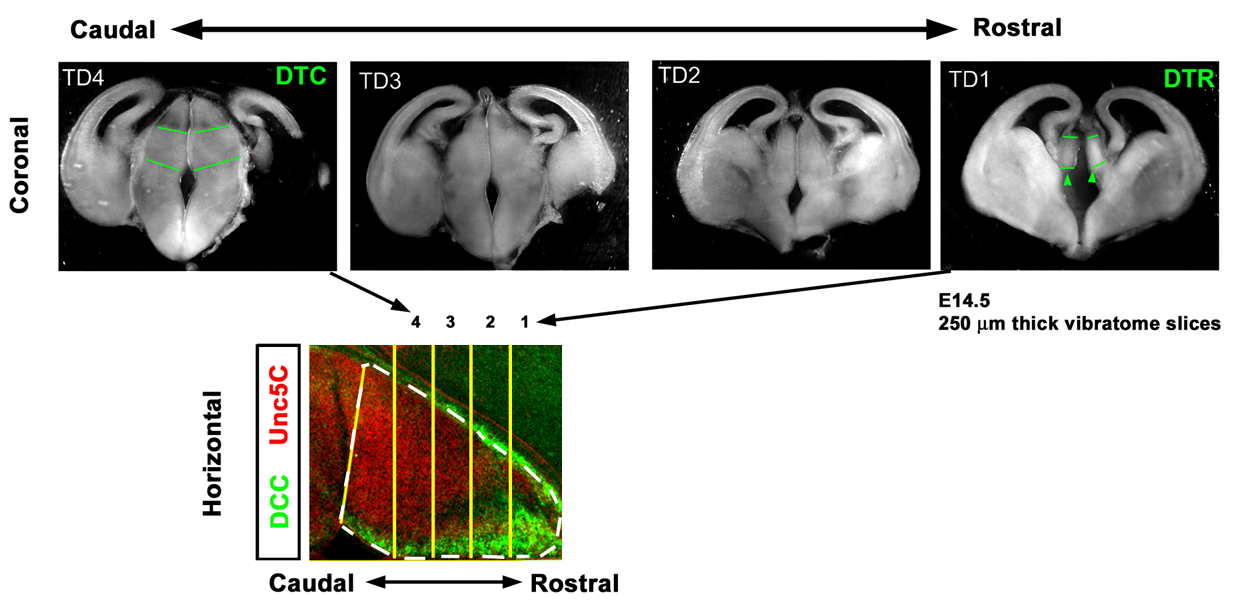

Supplement: Figure S11 — Dorsal thalamic explants are isolated on adjacent 250-μm–thick vibratome sections performed on E14.5 EGFP+ mouse embryos (top panel, ordered from rostral to more-caudal levels going from left to right). Expression patterns of DCC and Unc5C receptors, shown in Figures 7 and S10, suggest that section 1 (systematically used for DTR explants [10]) expresses high levels of DCC receptor, whereas section 4 (systematically used for DTC explants [10]) expresses low levels of DCC and high levels of Unc5A–C. (440 KB TIF) [file pbio.0060116.sg011.tif]

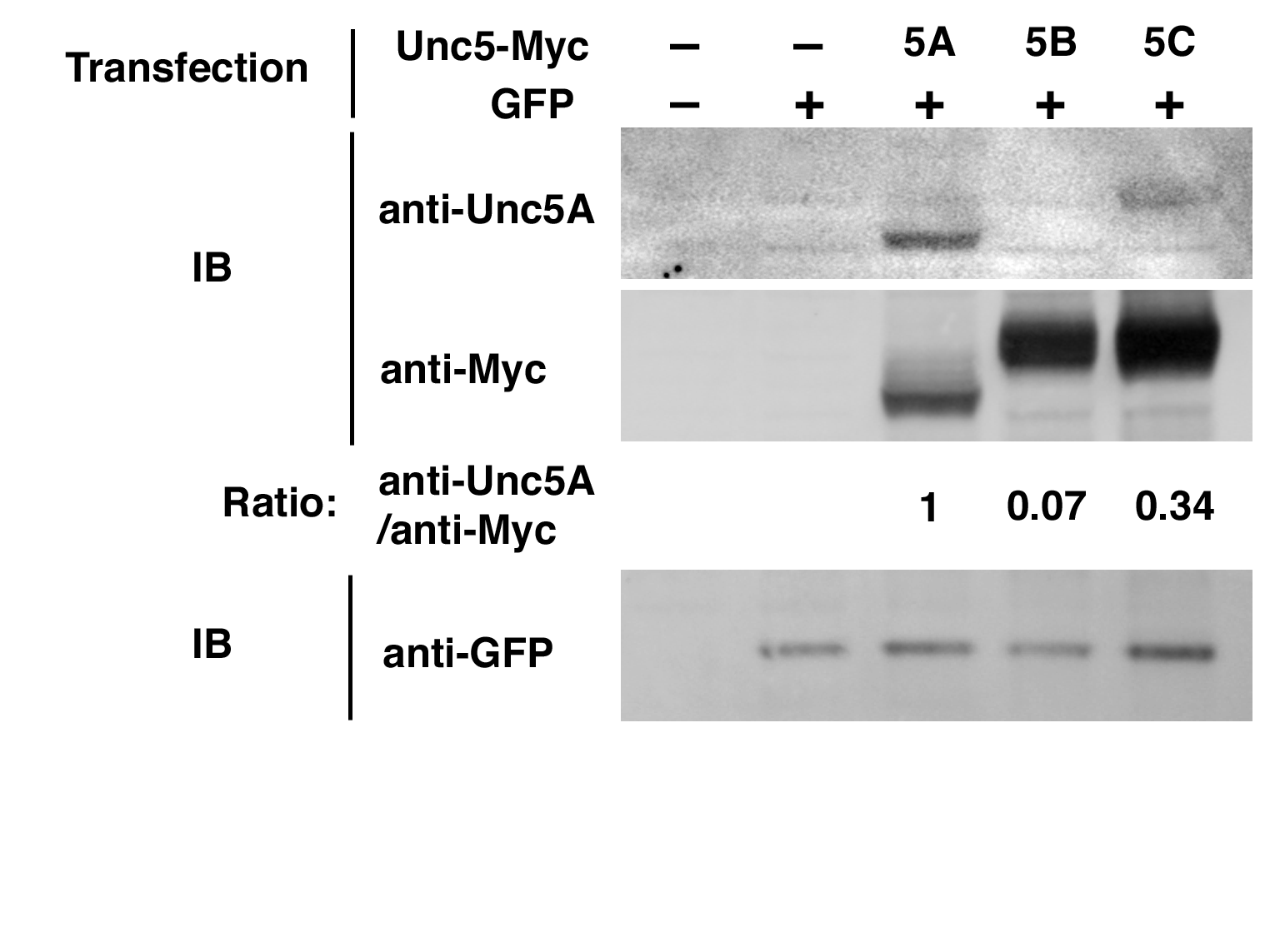

Supplement: Figure S12 — COS7 cells were transfected with myc-tagged Unc5A, Unc5B, or Unc5C expressed under chicken β-actin promoter (pCIG2) followed by IRES-mVenus. Two days after transfection, cells were lysed and lysates subjected to SDS-PAGE and probed with mouse anti-Myc antibody (clone 9B11, 1:2,000; Cell Signaling Technology), goat anti-Unc5A antibody (anti-rat Unc5H1, 1:200; R&D Systems), and rabbit anti-GFP antibody (IgG fraction, 1:2,000; Molecular Probes). The fluorescent signals were detected using Typhoon 9400 image scanner (Amersham) in the linear range. Ratios indicate the relative fluorescence obtained with anti-Unc5A (H1) and anti-myc (control for amount of recombinant Unc5A-B-C protein present in lysate). This analysis demonstrates that this commercially available anti-Unc5A cross-reacts with Unc5C (but not Unc5B) and has approximately three times more affinity for Unc5A than for Unc5C. (202 KB TIF) [file pbio.0060116.sg012.tif]
